# Deep Learning and Single-Molecule Localization Microscopy Reveal Nanoscopic Dynamics of DNA Entanglement Loci

**DOI:** 10.1021/acsnano.4c15364

**Published:** 2025-02-04

**Authors:** Maged F. Serag, Maram Abadi, Hajar Al-Zarah, Omar Ibrahim, Satoshi Habuchi

**Affiliations:** Biological and Environmental Science and Engineering Division, King Abdullah University of Science and Technology, Thuwal 23955-6900, Saudi Arabia

**Keywords:** DNA, entanglement, dynamics, deep learning, single-molecule localization microscopy

## Abstract

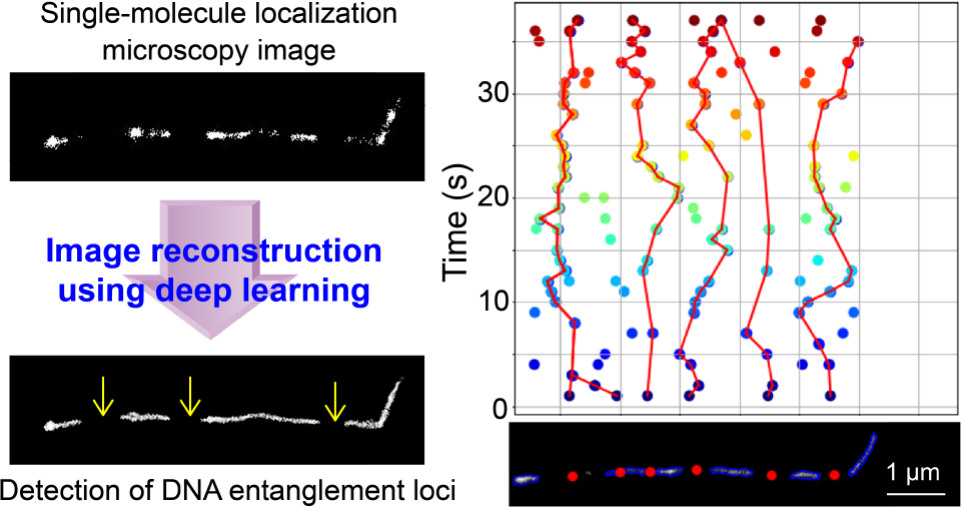

Understanding molecular dynamics at the nanoscale remains challenging due to limitations in the temporal resolution of current imaging techniques. Deep learning integrated with Single-Molecule Localization Microscopy (SMLM) offers opportunities to probe these dynamics. Here, we leverage this integration to reveal entangled polymer dynamics at a fast time scale, which is relatively poorly understood at the single-molecule level. We used Lambda DNA as a model system and modeled their entanglement using the self-avoiding wormlike chain model, generated simulated localizations along the contours, and trained the deep learning algorithm on these simulated images to predict chain contours from sparse localization data. We found that the localizations are heterogeneously distributed along the contours. Our assessments indicated that chain entanglement creates local diffusion barriers for switching buffer molecules, affecting the photoswitching kinetics of fluorescent dyes conjugated to the DNA molecules at discrete DNA segments. Tracking these segments demonstrated stochastic and subdiffusive migration of the entanglement loci. Our approach provides direct visualization of nanoscale polymer dynamics and local molecular environments previously inaccessible to conventional imaging techniques. In addition, our results suggest that the switching kinetics of the fluorophores in SMLM can be used to characterize nanoscopic local environments.

## Introduction

Deep learning has increasingly been applied to single-molecule localization microscopy (SMLM) techniques, such as Stochastic Optical Reconstruction Microscopy (STORM) and Photoactivated Localization Microscopy (PALM), to enhance spatial and temporal resolution. Numerous deep learning-based approaches have been developed, offering powerful tools for biological and biophysical research by improving throughput and functionality while addressing the limitations of traditional techniques.^1−9^

In SMLM, challenges often arise from the need to accumulate a large number of localizations (typically tens of thousands of frames) for a proper image reconstruction, which often leads to two primary challenges: photobleaching and poor temporal resolution.^10^ The deep learning tool Deep-STORM (Deep-Learning Stochastic Optical Reconstruction Microscopy) has emerged as a solution, employing a convolutional neural network for rapid, accurate, and parameter-free image reconstruction.^4,5^ Trained on simulated or experimental data, Deep-STORM facilitates the generation of single and multicolor high-resolution images with spatial resolution down to ∼19–31 nm while enabling real-time analysis. For an experimental microtubule data set of 500 frames containing ∼520,000 emitters, Deep-STORM achieves processing times of just 27 s using GPU acceleration, equivalent to localizing approximately 20,000 emitters per second. Similarly, ANNA-PALM (Artificial Neural Network Accelerated Photo-Activated Localization Microscopy)^6^ and FID-STORM (Fast dense Image reconstruction based on deep learning in STORM)^9^ reconstruct high-quality super-resolution images from sparse SMLM and widefield images, drastically reducing the required frames for reconstruction. ANNA-PALM achieves comparable resolution to standard PALM (∼20–30 nm) while reducing acquisition time by up to 2 orders of magnitude, reconstructing high-quality images from as few as 300–500 raw frames instead of 10,000–60,000. FID-STORM matches Deep-STORM’s resolution (52 nm for experimental microtubule data) while processing 256 × 256-pixel images at 137 frames per second, a 26-fold speed improvement. These artificial neural network (ANN)-powered approaches efficiently increase throughput and decrease imaging time and photobleaching while maintaining spatial resolution.

The significant enhancement in temporal resolution of SMLM, facilitated by deep learning, renders the study of relatively fast single-molecule dynamics, such as intracellular dynamics, feasible. The deep learning-based Single-Frame Super-Resolution Microscopy (SFSRM) method achieved high-fidelity live-cell imaging with 30 nm spatial and 10 ms temporal resolutions, enabling the observation of intricate intracellular dynamics of subcellular structures.^1^ Furthermore, deep learning has facilitated the measurement of single-molecule diffusion coefficients through motion blur analysis, eliminating the need for single-molecule localization or tracking. Pix2D (pixels-to-diffusivity) exemplifies this capability by directly determining diffusion coefficients from standard SMLM images, allowing for high-throughput, high-resolution diffusivity mapping of single molecules in lipid bilayers.^7^

Building upon this improvement in temporal resolution facilitated by deep learning in SMLM, we explored deep learning approaches to study entangled polymer dynamics at a fast time scale. In our previous study, we introduced an experimental method combining time-lapse super-resolution fluorescence localization microscopy with cumulative area (CA) tracking,^11−13^ enabling precise exploration of entangled polymer dynamics occurring in the time scale of 10 s. In this previous study, we determined DNA contours using a piecewise linear mapping approach applied to SR images, achieving the determination of their contours at the precision of 6–33 nm with 7–10 s of image acquisition. Using this method, we demonstrated the chain-position-dependent motion of the entangled chains, which is beyond the scope of the theoretical framework describing entangled polymer dynamics.^14,15^ Given the new scientific insight obtained in our previous study, SR imaging with even faster temporal resolution could unravel previously unknown entangled polymer dynamics.

In this study, we employed ANNA-PALM^6^ to produce SMLM images of fluorescently labeled Lambda DNA under entangled conditions with a temporal resolution of 0.5 s. To simulate the semiflexible DNA at entanglement conditions, we used the self-avoiding wormlike chain model with parameters accounting for the persistence length and excluded volume interactions of entangled DNA. We then generated simulated single-molecule localizations along these entangled DNA contours, varying parameters such as labeling density, localization precision, and noise levels. These simulated images were used to train the deep learning algorithm ANNA-PALM, enabling it to predict high-resolution DNA contours from sparse localization data under various experimental conditions. Unexpectedly, the analysis revealed dark segments in the reconstructed contours (i.e., areas that show a significantly lower localization density than other regions of the contours). Detailed analysis of the switching kinetics of the labeled dye molecule, Cy5, suggested that these segments are associated with entangled loci. Time-lapse analysis of the segments revealed stochastic and subdiffusive migration of the entangled loci. Our findings would advance the understanding of entangled polymer dynamics, particularly single molecule dynamics occurring at a fast time scale.

## Results/Discussion

### Simulation Lambda DNA Contours for Deep Learning Training and Their Validation

**Figure 1:**
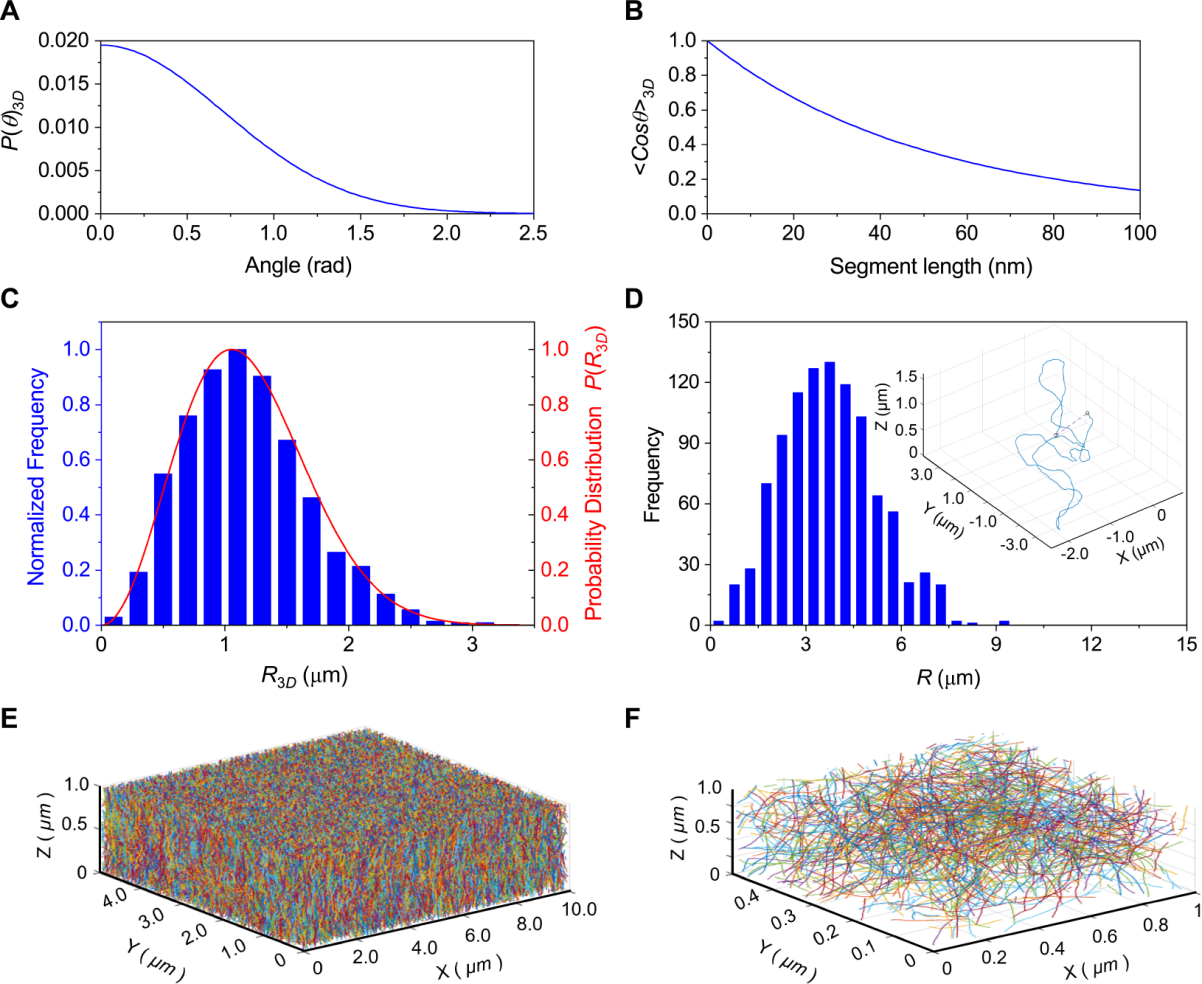
Simulation of Lambda DNA contours at entanglement. (A) The probability distribution of bending angles (*P(θ)*_*3D*_) in three dimensions calculated using eq 1. The DNA segment length was set at 50 nm. (B) Correlation between DNA segment length and bending Angle (θ) calculated using eq 2. (C) Frequency distribution of simulated end-to-end distance in three dimensions (blue bars). 2,500 molecules were used for the simulation. The red line shows the theoretically predicted probability distribution of end-to-end distance calculated using eqs 4 and 5 (D) Frequency distribution of end-to-end distance under the entangled conditions. Inset shows an example of a generated Lambda DNA 3D contour. The dashed line in the inset shows the end-to-end distance. (E) Simulation of entangled Lambda DNA matrix. (F) Enlarged view of the entangled DNA matrix shown in (E).

We aimed to leverage the computational strategy ANNA-PALM to study the dynamics of entangled Lambda DNA at rapid time scales. To achieve this, we generated a data set of simulated Lambda DNA contours to facilitate the training of the ANN. To simulate the contour of Lambda DNA, we used the semiflexible polymer Worm-Like Chain (WLC) model where the probability distribution of bending angles (*P(θ)*_*3D*_) is given by eq 1 (Figure 1A, segment length (*L*) = 50 nm) and the correlation between segment length (*L*) and bending Angle (θ) is given by eq 2 (Figure 1B).^16^ Using generated random values of segment lengths and bending angles, we iteratively constructed the Lambda DNA contour (contour length of Lambda DNA (*L*_*c*_) was set at 16.5 μm (48,502 bp)) by connecting segments with the specified lengths and orientations (Figure S1).
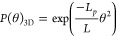
1
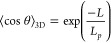
2
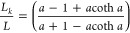
3where *L* is the theoretical segment length, *L*_*p*_ is the persistence length (*L*_*p*_ = 50 nm), *L*_*k*_ is the Kuhn length, and *a* is the bending constant (*a* = 50 nm).^17^

To ensure that the simulated DNA does not overlap with itself, we used the self-avoiding wormlike chain (SAWLC) model^17^ in conjunction with the previous distributions (eqs 1–3). By setting the effective width of the chain at ω = 4.6 nm,^17^ the algorithm allowed us to generate the DNA contour while accounting for the effects of excluded volume (see Supporting Methods).

To validate the accuracy of our Lambda DNA contours, we generated 2,500 simulated Lambda DNA molecules and computed the distribution of their end-to-end distances (*R*). We then compared this distribution with the theoretical expectations^18,19^ (eqs 4, 5, Figure 1C). Our analysis revealed a good agreement between the simulated and theoretical distributions, where the mean squared end-to-end distance calculated from the simulated data was 1.62 μm,^2^ which closely matches the theoretical value of 1.64 μm.^2^
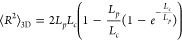
4
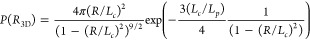
5

### Simulation of Lambda DNA Contours at Entanglement and Their Validation

To simulate the semiflexible DNA at entanglement conditions,^20^ we used eqs 6 and 7 to calculate the persistence length of entangled dsDNA (*L*_*pe*_ = 778 nm) with an entanglement length (*L*_*e*_) of 100 nm.^11^
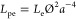
6
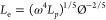
7where Ø is the volume fraction (Ø = 0.0059, see Supporting Methods). To validate the accuracy of our Lambda DNA contours, we generated 1,000 simulated Lambda DNA molecules and computed the mean end-to-end distances^21−24^ by using eqs 8-10
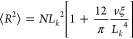
8where *N* is the number of Kuhn units, *v* is the excluded volume, and ξ is the correlation length.
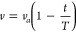
9where *v*_*a*_ is the excluded volume in athermal solvent (*v*_*a*_ = ω × *L*_*k*_), *t* is theta temperature (*t* = 14.7 °C,^25^ and *T* is the temperature of the system.
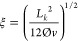
10

The calculated end-to-end distance was determined to be 5.7 μm. This value closely aligns with the simulated value of 5 μm (Figure 1D). The inset of Figures 1D and S2 illustrate the generated 3D contours of Lambda DNA at entanglement and their 2D projections.

We then simulated the generated Lambda DNA molecules within a matrix matching our experimental conditions (10 μm × 5 μm × 1 μm), with cubic voxels sized at 5 nm to match the effective width of the excluded volume. Utilizing the self-avoiding wormlike chain model and considering the excluded volume, we iteratively generated consecutive DNA segments for each molecule, as shown in Figure 1E,F (see Methods/Experimental section).

### Simulation of Localization Microscopy Images of DNA Contours for Deep Learning Training

**Figure 2:**
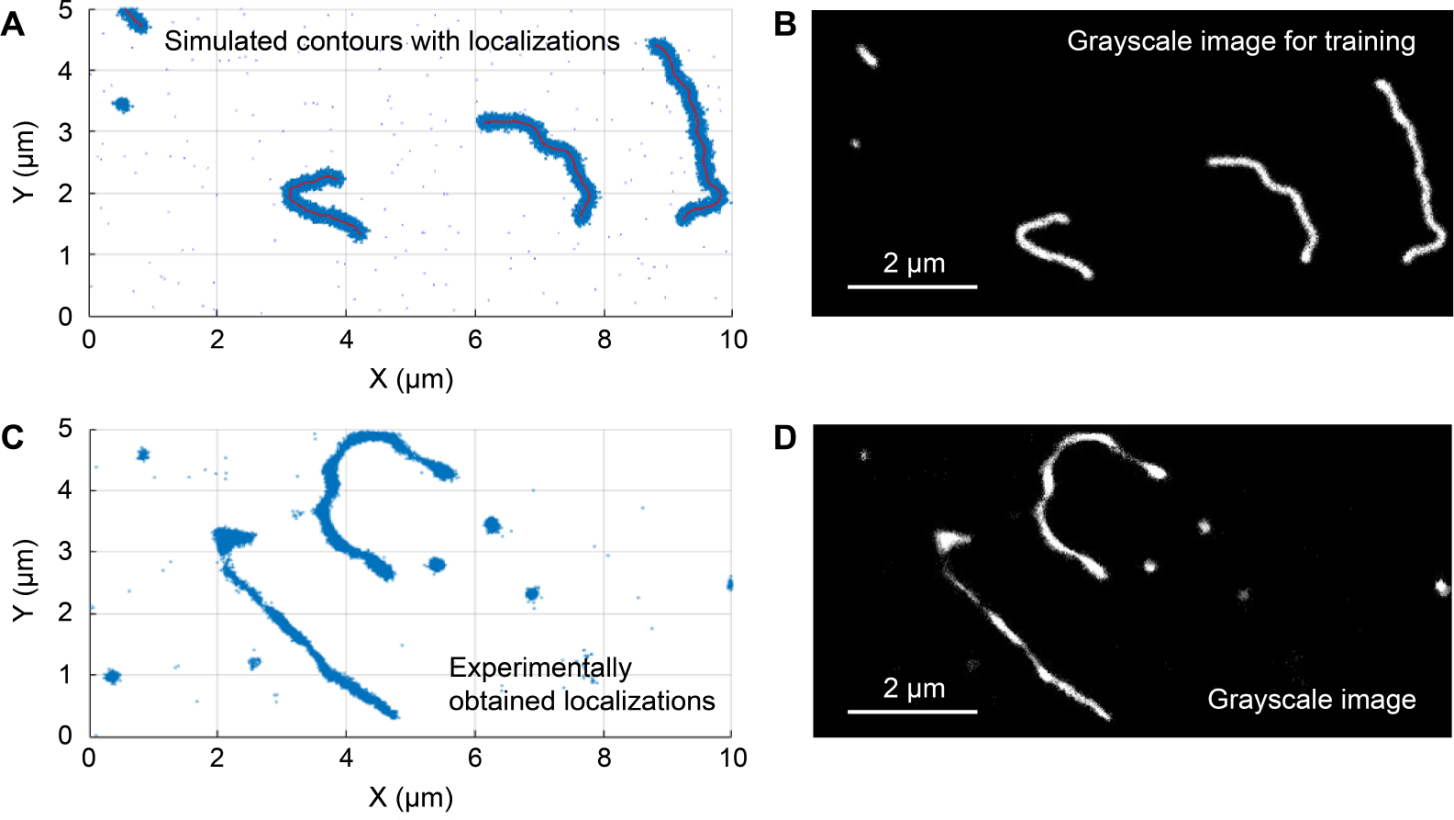
Simulated and experimental DNA contours in DNA matrix. (A) Simulated DNA contours (red lines) randomly highlighted in the DNA matrix and simulated localizations with image noise (blue dots, localization precision of 13 nm, and noise level of 1%). (B) 8-bit grayscale image generated from the localizations shown in (A). (C) Experimentally obtained SMLM image of Cy5-labeled Lambda DNA in the unlabeled matrix. (D) 8-bit gray scale image generated from the localizations shown in (C).

To simulate localizations derived from single-molecule switching for super-resolution imaging, we aimed to determine the number of localizations to simulate from each emitter and the distribution of these emitters (see Methods/Experimental section and Supporting Methods). Our calculations and experimental conditions indicated a labeling density of one dye molecule per 5–15 bp, with approximately 5 localizations for each individual dye molecule. According to our experimental findings, the distribution of these localizations around the emitters exhibits a standard deviation of 13 nm^11^ (Figure S3). We used these parameters to generate 3D localizations along randomly picked entangled Lambda DNA simulated contours (Figures 1E,F and 2A,B). The simulated SMLM image looks similar to the experimentally obtained SMLM image of Cy5-labeled Lambda DNA under entangled conditions (Figure 2C,D). We then used the 2D projection of these localizations to generate the required images for deep learning training, as shown in Figures S4 and S5.

### Predicting DNA Contours from Simulated SMLM Subsets and Evaluating Performance across Imaging Conditions

**Figure 3:**
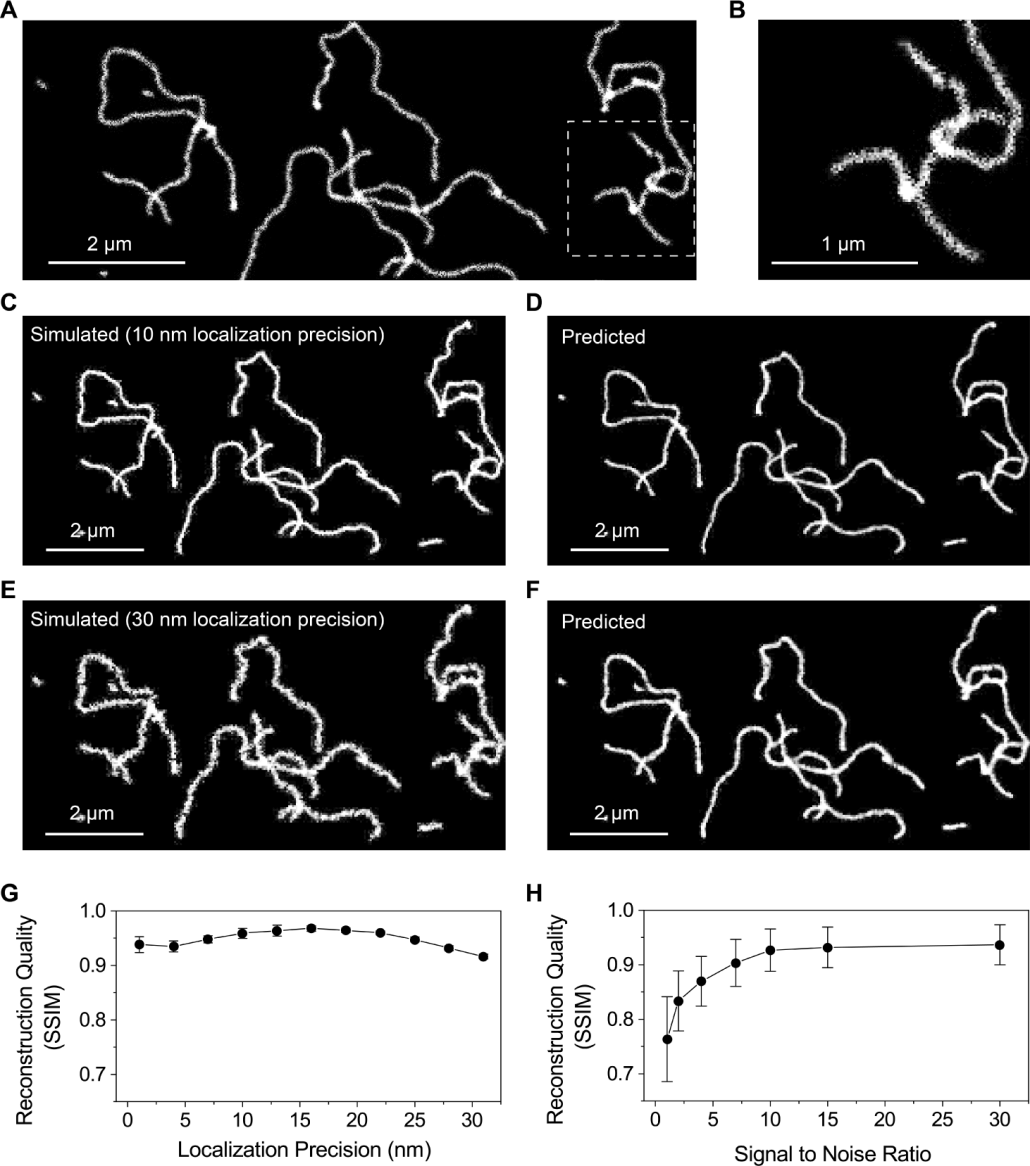
Simulation and Prediction of DNA contours under various imaging conditions. (A) Simulated DNA contours showing isolated and entangled molecules. (B) Enlarged view of the area highlighted by the dotted square in (A). (C, E) Simulated contours of Lambda DNA generated with localization accuracies of (C) 10 nm and (E) 30 nm. (D, F) Predicted images of the DNA contours simulated in (C) and (E). (G, H) Quantitative assessment of image prediction quality using the Structural Similarity Index (SSIM) under different levels of (G) localization accuracies and (H) signal-to-noise ratios (SNR). The error bars in g and h represent the standard deviation of 10 simulations.

Utilizing the simulated SMLM images, we conducted the training of ANNA-PALM with varying densities of DNA molecules within the imaging field to enable the prediction of both visually isolated and overlapping DNA molecules. This approach allowed us to ensure that our training model can handle scenarios where molecules are either visually isolated (the focus of our study) or entangled, as depicted in Figure 3A,B. To assess the robustness of our training and prediction, we tested the predicted DNA contours under various image quality conditions, specifically different levels of localization accuracies and noise present in the microscopy images. Figure 3C,E show simulated contours of Lambda DNA generation with localization accuracies of 10 and 30 nm, respectively. Their predicted DNA contours are displayed in Figure 3D,F. To rigorously assess the predictive capabilities of deep learning, we introduced random noise and noise originating from small DNA fragments within the imaging field. Additionally, we incorporated bright spots resulting from heavily labeled small DNA fragments into the simulations (Figure S6). It is important to note that we validated the robustness of the ANNA-PALM algorithm to detect DNA contours using uniform random noise (see below), which realistically simulates randomly dispersed fluorophores and DNA fragments typically present during single-molecule imaging.

To quantitatively evaluate the structural similarity between the simulated and predicted images, we employed the Structural Similarity Index (SSIM). Our analysis showed that, in the absence of noise, variations in localization accuracy have a relatively minor impact on the quality of generated images. Localization accuracies ranging from 1 to 30 nm consistently yielded SSIM values above 0.9 (Figure 3G; the usual SSIM benchmark of 0.8–0.9 for assessing image prediction quality.^26^ However, as the signal-to-noise ratio (SNR) varied from 1 to 30, SSIM values gradually increased, starting from a lower value of 0.7, reaching 0.9 when the SNR exceeded a threshold of 5 (Figure 3H).

We then assessed the deep learning algorithm’s performance in constructing DNA contours with varying levels of uniform random noise, localization densities of 1–3 localizations (i.e., 1–3 blinking events per fluorophore) per fluorophore, and labeling densities of one label every 5–50 bp. The noise level varied from 0% – 20%, where 20% represented random noise localizations, and the remaining localizations represented simulated positions along the contour (Figures S7–S9). Notably, all predicted images generated by the deep learning algorithm with one localization per fluorophore and with one fluorophore every 50 bp yielded SSIM values below 0.9, indicating the construction of the DNA contours with lower accuracy (Figure S7). Subsequently, we increased the fluorophore density to match the manufacturer-specified labeling conditions (one label every 5–15 bp). This adjustment resulted in reliable contour predictions (SSIM > 0.9) for noise levels up to 1% of the total number of localizations (Figure S8). Further increasing the number of localizations per fluorophore to 3 yielded reliable predictions even with up to 20% random noise (Figure S9). These results were obtained with a mean localization accuracy of 30 nm and an overall distribution width of 30 nm around the contour, beyond which predictions became unreliable. Increasing the distribution width to 60 nm, possibly due to local chain movement, led to predicted images displaying double contours, as each DNA chain was identified as a double chain due to the broader localization distribution (Figures S10, S11). To assess the temporal resolution of our analytical approach, we analyzed surface-deposited DNA molecules with known contours. We successfully reconstructed DNA contours from localizations accumulated at intervals as short as 0.5 s (500 sparse localization frames). The reconstructed contours showed good agreement with the expected DNA length over a 20-s period, demonstrating our method’s capability to capture DNA dynamics at subsecond time scales (Figures S12, S13, see Supporting Note 1).

Taken together, our results indicate that predictions can be considered 90% accurate under the following conditions: maximum mean localization accuracy of 30 nm, maximum distribution of localization accuracies of 30 nm, maximum noise percentage of 10%, minimum labeling density of 1 fluorophore every 15 bases, and a minimum total number of localizations allowing for 3 localizations per fluorophore. These parameters offer the necessary insights into the reliability and robustness of our prediction results for predicting DNA contours from super-resolution microscopy. Our experimental imaging conditions align with the criteria mentioned above, where we processed images with an average localization accuracy of 10–15 nm. We selected DNA contour areas distant from noise to minimize noise interference, as detailed in our prior publication.^11^ Under these minimized noise conditions, ANNA-PALM accurately reconstructs contours even with sparse localizations, achieving SSIM values above 0.9 with one localization per fluorophore (Figure S8B). Furthermore, our experimental results indicated an average of 2.8–8.7 localizations per dye molecule (see the calculations in the Methods/Experimental section and Supporting Methods). Thus, we conclude that we generated reliable predicted DNA contours (see Supporting Note 1).

### Nonrandom Spatial Distribution of the Localization along the Contours

We obtained SMLM images of entangled Lambda DNA by adding a small amount of fluorescently labeled DNA into the nonlabeled matrix DNA in the switching buffer. We labeled the DNA with Cy5 dyes at the labeling density of 5–15 bp per dye molecule. The final concentration of the mixed DNA was 5–10 mg mL^–1^ (see Methods/Experimental section for the experimental details). Single-molecule fluorescence images of the mixed DNA sample were recorded at 1 kHz for 65 s, from which we reconstructed SMLM images.

**Figure 4:**
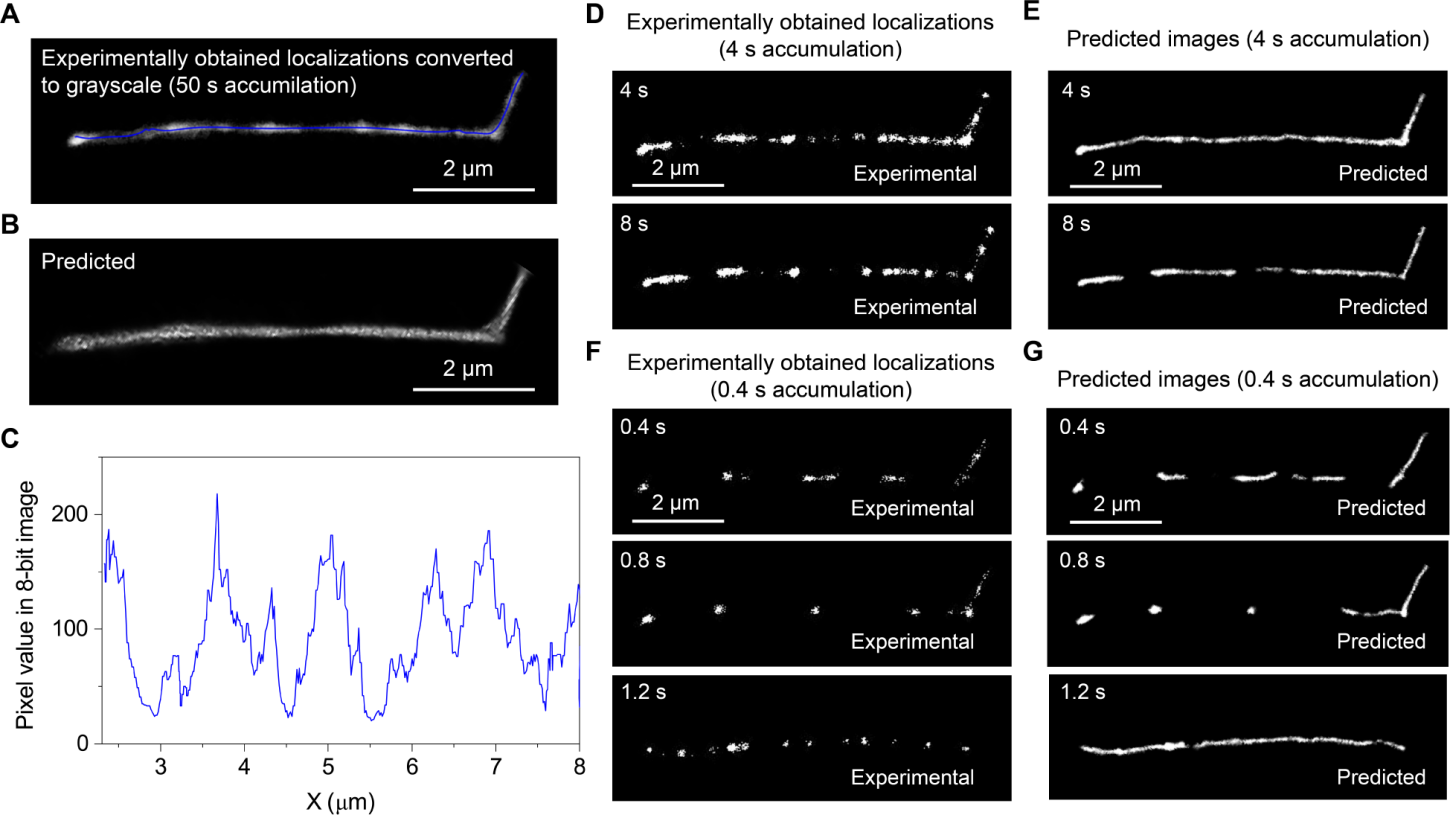
DNA contour prediction at varying imaging time scales. (A) SMLM image of Lambda DNA reconstructed using single-molecule images acquired over 50 s, and converted to an 8-bit grayscale image. (B) Predicted DNA contour from the experimental data shown in (A). (C) Intensity profile along the DNA contour displayed in (A) (blue line). (D, F) Time-lapse SMLM images of Lambda DNA reconstructed using single-molecule images acquired during (D) 4 s and (F) 0.4 s (see Figure S18 for more examples). (E, G) Predicted DNA contours from the experiments shown in (D) and (F).

Figure 4A shows an experimentally obtained SMLM image reconstructed using the images captured over 50 s (i.e., 50,000 frames). The predicted image generated by ANNA-PALM exhibited a continuous contour of Lambda DNA (Figure 4B), demonstrating the successful prediction of DNA contour. The predicted DNA contour showed inhomogeneous width. This finding can be interpreted by the local motion of the DNA during the 50-s image acquisition (Figures S10, S11).^11^ Importantly, the intensity profile along the DNA contour reveals distinct peaks separated by multiple minima, where the maxima in the profile are 8 times greater than the minima (Figure 4C). This pattern indicates significant spatial variability in the distribution of localizations along the DNA contour. The observed spatial distribution of the localizations is not a result of stochastic on–off switching of the Cy5 dyes, as Cy5-labeled Lambda DNA deposited on a surface covered by the switching buffer showed a more random distribution of the localizations along the contour (Figure S14).

The inhomogeneous distribution of the localizations is more apparent in SMLM images reconstructed with a smaller number of frames. The SMLM images reconstructed using the single-molecule images captured over 4 s exhibited small gaps (i.e., dark segments) along the contours (Figure 4D). The dark segments become more significant in the SMLM with 0.4 s image acquisition time (500 sparse localization frames, Figure 4F). Two factors contribute to the dark segments observed in the reconstructed images. First, the stochastic switching of the Cy5 dyes results in a random spatial distribution of the localization. At a short image acquisition time, the total number of localizations in a reconstructed image is small (i.e., lower localization density), resulting in dark segments. This effect is more significant in 0.4 s image acquisition than in 4 s acquisition (Figure 4D,F). Since ANNA-PALM can efficiently handle the gap regions caused by the nature of stochastic switching, some gaps are nicely filled in the predicted contours (Figure 4E,G). The predicted contour obtained at 1.2 s time point demonstrates that we could reconstruct the contours of entangled Lambda DNA molecule with the temporal resolution of 0.4 s, more than an order of magnitude faster than that in our previous study.^11^

Another factor contributing to the observed dark segments is the nonrandom distribution of the localizations along the contour, indicated in Figure 4C. The dark segments associated with this appear as gaps along the contours in the predicted images generated by ANNA-PALM because the algorithm was trained with the random spatial distribution of the localizations along the contours. Thus, the predicted contours highlight the dark segments along the contours associated predominantly with the nonrandom distribution of the localization.

### Local Diffusion Barriers Cause the Nonrandom Distribution of the Localization

The SMLM imaging on the entangled Lambda DNA and the contour prediction by ANNA-PALM revealed the dark segments along the contours caused by the nonrandom distribution of the localization. We note that the pattern of alternating bright and dark segments was observed in reconstructed SMLM images of labeled DNA deposited on a surface on which an unlabeled DNA matrix was overlaid (Figure S15). The presence of the pattern in this two-dimensional sample suggests that the dark segments (Figure 4) cannot be attributed to the three-dimensional looping of DNA, where specific regions may exist out of focus, although we cannot completely exclude its contribution to the observed dark segments.

**Figure 5:**
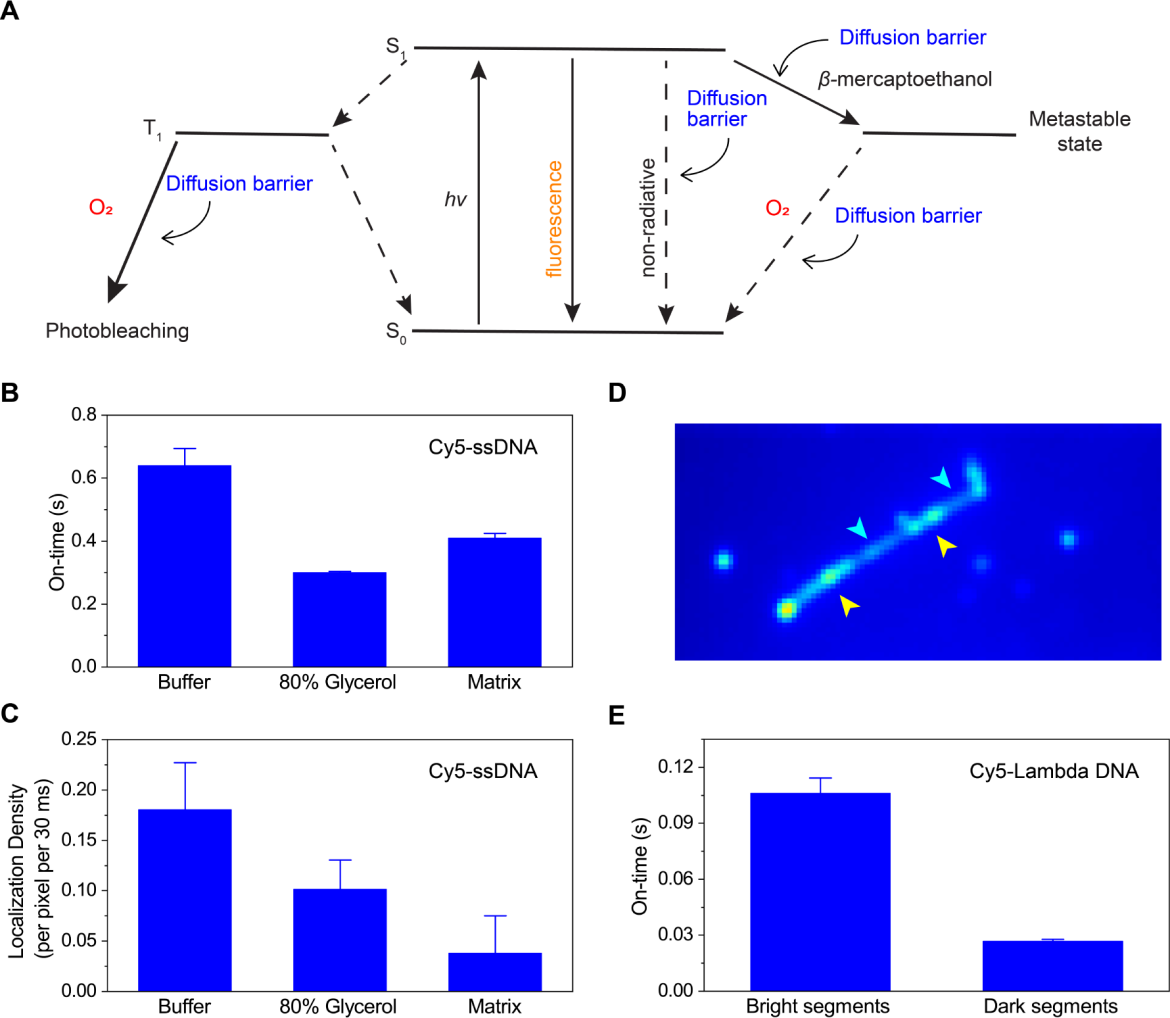
Effect of viscosity on on–off switching of Cy5 fluorescence. (A) Jablonski diagram showing photophysical pathways of Cy5. Upon light absorption (*hν*), electrons transition from the ground state (S_0_) to the excited state (S_1_), followed by fluorescence emission when returning to S_0_. From S_1_, electrons can transit to a triplet state (T_1_), where oxygen-mediated oxidation leads to irreversible photobleaching (left pathway). Local diffusion barriers can restrict O_2_ accessibility, reducing photobleaching rates. Alternatively, β-mercaptoethanol can cause electron transition to a metastable dark state (right path), a process that can be inhibited by diffusion barriers. The return to S_0_ from this dark state is facilitated by O_2_, which is also affected by local diffusion barriers, modulating the overall photoswitching kinetics. (B) Average fluorescence on-time of Cy5 conjugated to ssDNA obtained in the switching buffer without glycerol (left), with 80% glycerol (center), and with DNA matrix (right). The error bars show the standard errors in the fitting of the on-time distributions to an exponential decay function displayed in Figure S16. (C) Average density of single-molecule localizations of Cy5 conjugated to ssDNA obtained in the switching buffer without glycerol (left), with 80% glycerol (center), and with DNA matrix (right). The error bars show standard deviations of time-dependent localization densities. (D) Example of the predicted image of Cy5-labeled Lambda DNA in the unlabeled matrix. Bright and dark segments are highlighted by yellow and cyan arrows, respectively. (E) Average fluorescence on-time of the Cy5 molecules conjugated to Lambda DNA in the bright and dark segments obtained in the switching buffer with DNA matrix. The error bars show the standard errors in fitting the on-time distributions to an exponential decay function displayed in Figure S17.

The excited-state photochemical reaction between the Cy5 dyes and the reducing reagent in the switching buffer (β-mercaptoethanol) causes the on-to-off switching of Cy5 (Figure 5A). Oxygen-mediated off-to-on switching, as well as photobleaching, also affect the fluorescence switching behavior of Cy5 (Figure 5A).^27^ Since both the excited state redox reaction and the oxygen-mediated processes are affected by the local molecular environment, the presence of diffusion barriers around the Cy5 dyes could cause position-dependent switching kinetics, which may result in the nonrandom distribution of the localization. In addition, the excited-state lifetime of cyanine dyes is significantly affected in environments with restricted molecular motion, such as those created by entangled DNA,^28^ which would also influence the overall switching behavior of the Cy5 dyes and, thus, the distribution of the localization. These local diffusion barriers, created by DNA entanglement, can impede the movement of switching buffer components, leading to heterogeneous reaction environments along the DNA contour.

To investigate the effects of diffusion barriers on fluorophore behavior, we examined the fluorescence switching kinetics of the surface-deposited Cy5 molecules conjugated to 50 nt ssDNA in the switching buffer with varied viscosity (see Methods/Experimental section and Supporting Note 2 for experimental details) and compared the obtained results with the switching kinetics observed for the Cy5 dye-labeled DNA in the nonlabeled Lambda DNA matrix. By modulating the viscosity of the medium, we could experimentally simulate the hindered diffusion of switching buffer molecules. This approach allowed us to compare the effects of viscosity-induced diffusion hindrance with those observed in the entangled DNA matrix, where molecular entanglements create physical barriers to diffusion. Two switching buffers with different viscosities were prepared by mixing the aqueous buffer with glycerol. The viscosities of the buffers were adjusted to 1 mPa s (0% glycerol) and 80 mPa s (80% glycerol) (see Method/Experimental section for the determination of the viscosity). We note that the viscosity of the switching buffer with 80% glycerol (80 mPa s) is similar to the macroscopic viscosity of the Lambda DNA matrix (78 mPa s).

The viscosity-dependent fluorescence on-time distribution shows that the Cy5 dyes exhibit shorter on-times in the higher viscosity media compared with that in the lower viscosity (Figures 5B and S16). We cannot fully explain this on-time behavior, but it is plausible that the on-time is determined by the balance between oxygen scavenging efficiency and β-mercaptoethanol-mediated excited-state reduction of Cy5 dyes. Notably, the single-molecule localization density of the Cy5 dyes (i.e., the number of localizations per unit time) follows a similar trend (Figure 5C). We observed the lower localization density in the higher viscosity media compared with the medium with the lower viscosity (Figure 5C). Together, our findings demonstrated that the Cy5 dyes display shorter on-time with smaller localization density in the higher viscosity (i.e., 80 mPa s) environments compared with those in the environments with the lower viscosity (i.e., 1 mPa s).

Next, we analyzed the on-time distribution of the Cy5 molecules in the bright (i.e., high localization density) and dark (i.e., low localization density) segments of the Cy5-labeled Lambda DNA in the matrix DNA identified by ANNA-PALM (Figure 5D). The mean on-time of the bright and dark segments were 0.11 and 0.027 s, respectively (Figures 5E and S17, Supporting Note 3). The result demonstrates that the Cy5 molecules in the lower localization density regions have shorter on-time than those in the higher localization density regions. While the macroscopic viscosity of the switching buffer with 80% glycerol (80 mPa s) matches that of the DNA matrix (78 mPa s), their local environments differ significantly. Analysis of the on-time distributions reveals that bright segments show similar on-times (0.11 s, Figure S17A) to surface-deposited DNA in switching buffer (0.10 s, Figure S17C), suggesting minimal diffusional barriers in these regions. We also note that the relationship between viscosity and Cy5 fluorescence is complex, influenced by multiple factors including on-time, quantum yield, and photoswitching kinetics. These competing effects result in nonlinear brightness changes with viscosity, as evidenced by shorter fluorescence on time in 80% glycerol compared to lower viscosities. Taken together and given the correlation between the viscosity of the media and the on-time and localization density observed for the ssDNA-conjugated Cy5 molecules, the finding strongly suggests that the nonrandom distribution of the localization along the contours of the Cy5 labeled Lambda DNA is associated with a spatial variation in the local diffusional properties.

### Visualization of Entanglement Loci along the Entangled DNA

**Figure 6:**
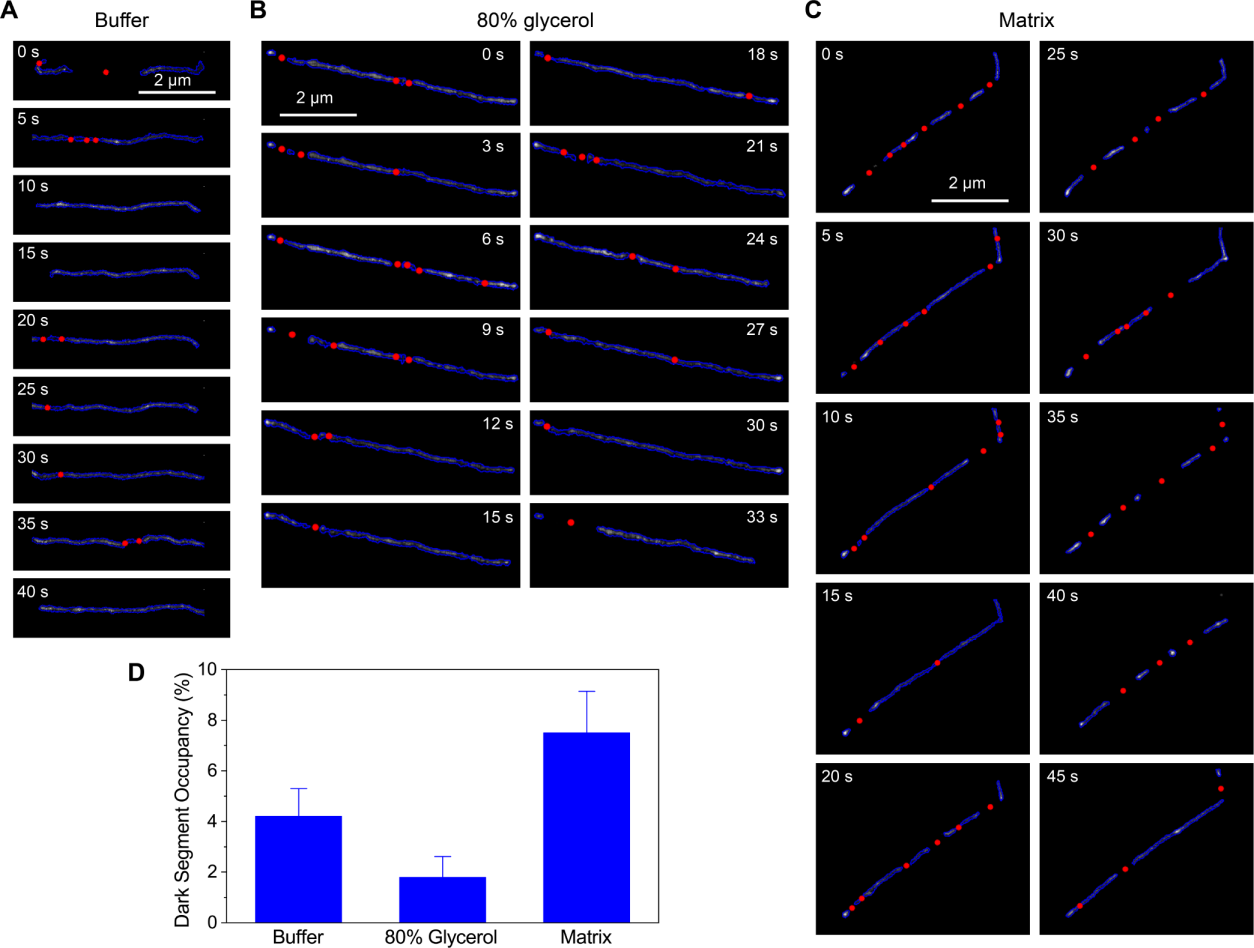
Predicted DNA contours and dark segment centers in various imaging conditions. (A–D) Time-lapse images of the predicted DNA Contours (blue lines) and the centers of the dark segments (red circles) obtained in the switching buffer (A) without glycerol, (B) with 80% glycerol, and (C) with DNA matrix. Each SMLM image was reconstructed using single-molecule images acquired during 1 s. (D) Percentages of the dark segments along the DNA contours. The error bars in D show the standard deviation of 46 molecules in the matrix, 32 molecules in 80% glycerol, and 31 molecules in the buffer. See Figure S18 for more examples of Lambda DNA embedded in a DNA matrix.

We then examined whether the dark segments observed in the predicted SMLM images of the entangled DNA by ANNA-PALM (Figure S18) are predominantly associated with the variation in the local molecular environment created by differences in entanglement density. To that end, we conducted an SMLM imaging experiment of the Cy5-conjugated Lambda DNA in the switching buffer with different viscosity. We captured single-molecule images of Cy5 dyes with 1 ms exposure time and reconstructed an SMLM image using the single-molecule images captured during the period of 1 s (i.e., 1,000 frames) (see Methods/Experimental section for experimental details). The time-lapse images of the predicted SMLM images by ANNA-PALM are displayed in Figures 6A-C and S19– S21. In each time-lapse image, the contours of the Lambda DNA and the center position of the dark segments are denoted by blue lines and red circles, respectively (Figure 6A-C). We note that our method reliably detects dark segments extending beyond 300 nm (see Methods/Experimental section and Supporting Note 4). Dark segments smaller than 300 nm are not detected by ANNA-PALM, as the algorithm always predicts the contour (i.e., fills these gaps).

The predicted time-lapse SMLM images of the Lambda DNA by ANNA-PALM in the homogeneous solution (i.e., Lambda DNA in the switching buffer with glycerol) exhibit a small fraction of dark segments (Figure 6A,B,D). The results indicate that a small fraction of the dark segments appearing in the predicted images of the Lambda DNA in the homogeneous solutions are associated with the stochastic switching behavior of the Cy5 dyes. The predicted time-lapse SMLM images of the Lambda DNA by ANNA-PALM in the DNA matrix show the more frequent and much larger fraction of the dark segments (Figure 6C,D). Notably, while the matrix and 80% glycerol samples exhibited similar on-time (i.e., similar average viscosity, Figure 5B), the fraction of the dark segments was significantly higher in the matrix (Figure 6D). These results suggest that most of the dark segments observed for the Lambda DNA in the DNA matrix are associated with the nonrandom spatial distribution of the localization. In addition, the dark segments observed for the Lambda DNA in the DNA matrix are persistent (i.e., appearing at close positions along the contour in consecutive time-lapse images; see Methods/Experimental section, discussion below on tracking of dark segments, and Supporting Note 5), which is not the case for the Lambda DNA in the homogeneous media (Figure 6A,B). Given the critical contribution of the local environment to the localization density, these results suggest that the predicted time-lapse SMLM images of the Cy5-conjugated Lambda DNA in the matrix capture the variation of the effective entanglement density along the contour of the entangled DNA (see Supporting Note 6). Since the difference between the Lambda DNA in the switching buffer with 80% glycerol and in the switching buffer containing the matrix DNA is the presence of surrounding entangled Lambda DNA molecules, the spatial variation observed for the entangled DNA indicates the spatial variation of the entanglement along the contour (i.e., presence of entanglement loci) that acts as a structural diffusion barrier.

### Characterization of Entanglement Loci Dynamics

**Figure 7:**
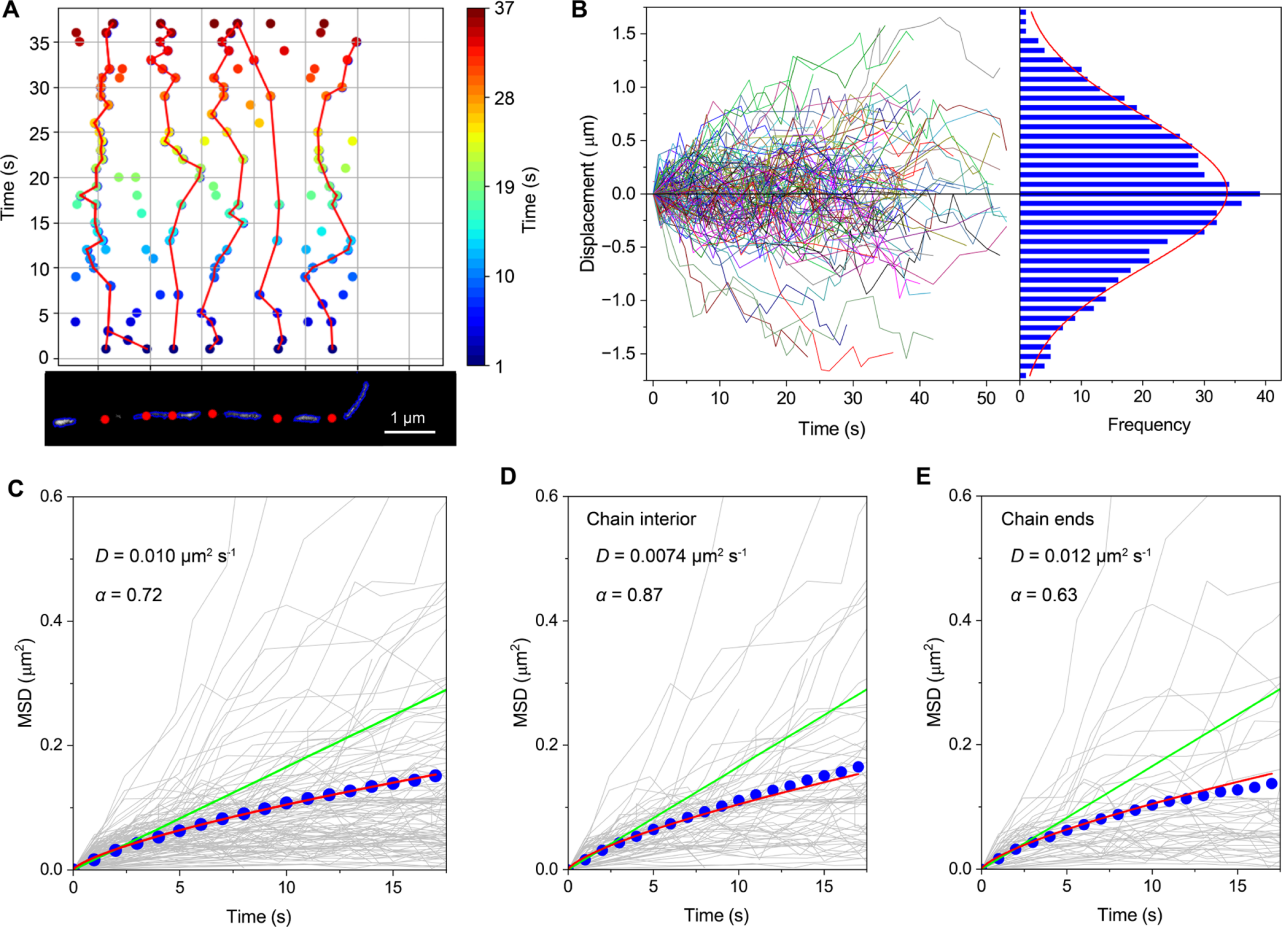
Dynamics of DNA entanglement loci. (A) Temporal dynamics of dark segments’ centers obtained from the predicted image of Cy5-labeled Lambda DNA in the unlabeled matrix. (B) Time-dependent displacement of the center positions of the dark segments obtained from 100 trajectories (left), and the distribution of the displacement (right, blue bars) fitted to a Gaussian function (right, red line). (C–E) Mean squared displacement (MSD) plots versus time lag obtained from the individual time trajectories of the center positions of the dark segments (gray lines); trajectories obtained from the (C) entire positions, (D) interior region (64 dark segments), and (E) end region (51 dark segments) along the contours. For chains with at least three dark segments, we classified the terminal segments closest to chain ends as “chain ends” while the remaining segments between these were classified as “chain interior.” The MSD plots shown in blue are the average of individual tracks. The green lines show the calculated MSD plots for a random displacement model with diffusion constants obtained from the initial slope of the plots. The red lines show the MSD plot fitted to the subdiffusion model (MSD = 2*Dtα*).

The connection between the dark segments and the entanglement loci acting as topological constraints indicated by the reconstruction of the predicted SMLM images of the Cy5-conjugated Lambda DNA in the nonlabeled matrix DNA by ANNA-PALM (Figure 6C, red circles) enables us to characterize spatiotemporal dynamics of the entanglement loci. To quantify their dynamics, we tracked the centers of these dark segments (see Methods/Experimental section for details) in the time-lapse SMLM images obtained at 1 s intervals. Figure 7A illustrates the temporal dynamics of the centers of the dark segments (Figures S22, S23, and Supporting Note 4), with the *x*-axis representing the position along the contour and the *y*-axis denoting time. The trajectories depict the variation in the x-position of these centers over time. Importantly, these trajectories are persistent throughout the image acquisition. This contrasts with the short and less-defined trajectories found for the Lambda DNA in the homogeneous viscous solution (i.e., 80% glycerol, Figure S24), which are caused by the occasional appearance of the dark segments due to the stochastic on–off switching behavior of the Cy5 dyes. The results suggest that the trajectories of the dark segments obtained for the entangled Lambda DNA capture the migration of the entanglement loci along the contour over time (Figure 7A). Figure 7B shows the time-dependent displacement of the entanglement loci obtained from 100 trajectories. The frequency distribution of the displacement was fitted well to Gaussian, with a peak at around 0 μm (Figure 7B), demonstrating a nondirectional motion along the contours. Figure 7C shows mean squared displacement (MSD) plots versus time lag obtained from the individual time trajectories of the center positions of the dark segments. The average MSD plot deviates from the random diffusion and exhibits a subdiffusive behavior, with a diffusion coefficient (*D*) of 0.010 μm^2^ s^–1^ and diffusion exponent (α) of 0.72. To investigate whether the entanglement loci dynamics is dependent on the position along the contour, the MSD analysis was performed on dark segments located near the end of the chains (Figure 7E) and those obtained from the center of the chains (Figure 7D). The resulting MSD plots showed similar subdiffusive behavior with similar *D* values for both regions (Figures 7D,E). These findings suggest that the behavior of entanglement loci is not position-dependent along the polymer chain.^11^

The entanglement number of Lambda DNA under the experimental condition (5–10 mg mL^–1^ concentration) is estimated to be 63–158 per chain.^11^ These numbers are much larger than the number of the dark segments captured in this study (3–4 per chain). The entanglement loci captured in this study are, thus, not caused by each entanglement. Instead, they capture spatially heterogeneous entanglement density along the contour and its change over time. The entanglement number of 63–158 per chain corresponds to one entanglement every 104–262 nm along the DNA contour. This is consistent with our finding that the Cy5 dyes in the bright segments exhibited on-times (Figure S17A) similar to that observed for the surface-deposited DNA in the switching buffer (Figure S17C), suggesting minimal diffusion barriers at this relatively low average entanglement density. The significantly shorter on-times in the dark segments (Figure S17B) that suggest the existence of substantial diffusion barriers can be interpreted by much higher local entanglement density in the entanglement loci.

Our previous study showed that the reptation motion (i.e., the motion of the chain along a virtual tube created by surrounding chains) of the entangled Lambda DNA (5–10 mg mL^–1^ concentration) occurs at the time scale of minutes to tens of minutes.^11^ We also showed in the previous study that local chain motion occurs in the seconds to minutes time scale under the entangled conditions, which was interpreted by the constraint release model of entangled polymer dynamics.^11^ The migration of the entanglement loci captured in this study occurs in the time scale of seconds, much faster than the reptation motion and close to the time scale of local chain motions.

Multichain simulations using various single- and multichain models have been the predominant approach to investigate the microscopic (i.e., subchain level) structure and dynamics of entangled polymers.^29−31^ In this study, although in an indirect way, we experimentally captured the motion of multiple chains surrounding the visualized chain. We observed position-independent dynamics of the entanglement loci (Figure 7D,E), aligning with assumptions in multichain polymer physics models like slip-link and slip-spring models that predict uniform chain interactions at entanglement points. The subdiffusive behavior of entanglement loci (α = 0.72, *D* = 0.010 μm^2^ s^–1^) could reflect constrained motion arising from topological interactions between multiple chains. In the simulation studies, while a distribution of the chain length between entanglements due to statistical variability has been reported,^32,33^ the presence of entanglement loci is not assumed. In this study, we experimentally showed spatially heterogeneous chain entanglement along the contour of the polymer chain beyond statistical distribution. These findings suggest the need for new theoretical frameworks incorporating spatially heterogeneous entanglement distributions.^30,31^ This study is the first step toward the experimental investigation of the microscopic entangled polymer dynamics, which may open up new possibilities for the experimental verification of the theoretical and simulation studies on the microscopic entangled polymer dynamics.

## Conclusion

In this study, we showcased the integration of artificial intelligence with advanced fluorescence microscopy to enhance nanoscale imaging and analysis capabilities. By combining deep learning with single-molecule localization microscopy, we have developed an approach that reveals the previously unobserved dynamics of DNA entanglement loci at the nanoscale with improved temporal resolution, while maintaining spatial precision needed for single-molecule tracking. We trained an artificial neural network (ANNA-PALM) to predict the contours of Lambda DNA under entanglement from simulated sparse localization frames. We demonstrated that this approach produced high-quality SMLM images of entangled chains with reduced acquisition times, enabling the exploration of fast dynamics. Using the experimental and analytical techniques developed in this study, we revealed bright (i.e., densely localized) and dark (i.e., sparsely localized) segments along the contours. The nonhomogeneous distribution of localizations along the DNA contour provided insights into polymer dynamics at the nanoscale where it is a direct result of polymer behavior, specifically the varying densities of entanglements along the DNA. The tracking of the dark segments indicated stochastic migration and subdiffusive behavior of these loci.

Our findings pave the way for a deeper understanding of entangled polymer dynamics and their implications in various biological and materials science applications. In addition, local environments quantified by SMLM in this study through the synergistic combination of deep learning, SMLM, and analytical tools could be utilized for nanoscopic characterization in diverse research fields, including polymer physics, biophysics, and materials science.

## Methods/Experimental

### Sample Preparations

Samples were prepared, as shown in our previous study.^11^ Briefly, Lambda DNA was precipitated using sodium acetate and isopropanol, washed with ethanol, and dissolved in TE buffer to obtain a 17 mg mL^–1^ solution. Fluorescence labeling was performed using a Mirus Bio kit, achieving 5–15 bp/dye labeling density by incubating Lambda DNA with Label IT Cy5 at 37 °C for 2 h. Labeled DNA was purified via ethanol precipitation and dissolved in TN buffer to 5 μg mL^–1^. The switching buffer contained an oxygen scavenging system (catalase, glucose oxidase, glucose) and 200 mM β-mercaptoethanol in TN buffer. For imaging, 5–10 mg mL^–1^ labeled DNA was mixed with unlabeled matrix DNA, NaCl, and switching buffer, then sandwiched between a coverslip and slide using a 0.12 mm adhesive spacer. The SMLM imaging experiment on suspended or deposited Lambda DNA or poly-Thymine 50mer ssDNA-Cy5 involved depositing fluorescently labeled DNA on a coverslip, mixing (for suspended samples) or overlaying (for deposited samples) it with switching buffer, 80% glycerol, or Lambda DNA matrix, and sandwiching it between the functionalized coverslip and a glass slide sealed with an adhesive.

### Fluorescence Imaging Experiment

We used a custom-built widefield epi-fluorescence microscope as described in our previous study^11,34^ with a 638 nm laser, acousto-optic tunable filter, achromatic focusing lens for Köhler illumination, 1.49 NA oil objective, dichroic, emission filters, and an EMCCD camera with OptoMask cropping. Images were captured using SOLIS software, with a C-Focus system controlling axial drift. Fluorescently labeled DNA was excited at <0.1 kW cm^–2^ to locate an isolated molecule in the field of view. Excitation was then increased to 25–30 kW cm^–2^ with a switching buffer to promote Cy5 switching when the density reached several fluorescent spots per image. 65,000 images were acquired at 1.0–1.4 kHz with a 100 × 50-pixel ROI, 100 nm pixels, and 300 EM gain. The configuration of 100 × 50 pixels makes use of the camera’s line-by-line readout, enabling faster frame rates than square regions of equivalent area. These rectangular images were processed to match ANNA-PALM’s required input dimensions (512 × 512 pixels).

Each SMLM image was reconstructed from 1,000 raw images (1 kHz frame rate). Illumination occurred only during acquisitions to minimize photobleaching. It is important to note that our imaging approach focused on DNA molecules oriented primarily horizontally within the imaging field. This selective imaging strategy was employed to maximize the observable contour length and increase the likelihood of capturing multiple entanglement points along a single molecule. This approach allowed us to quantify entanglement loci and gather statistics on a sufficient number of entanglement events, which would have been challenging with randomly oriented molecules in our 2D imaging.

### Estimating Labeling Density and Localization Distribution for Super-Resolution Imaging Simulations

To calculate the number of localizations to simulate from each emitter and the spacing between these emitters, we performed the following calculations: In the imaging field measuring 5 μm × 10 μm × 1 μm, there are approximately 9,600 DNA molecules, among which 11.2 are labeled (see Supporting Methods). With a labeling density of one dye molecule per 5–15 bp (as per the manufacturer specifications, this corresponds to a range of 3,233 to 9,700 dye molecules per Lambda DNA molecule (48,502 base pairs)). This translates to an estimated 35,563 to 106,700 dye molecules in the imaging field. With a total of 300,000 localizations, on average, there are approximately 5 localizations for each dye molecule. Based on our experimental data, the distribution of these localizations on both sides of the emitters has a standard deviation of 13 nm.^11^

### Generation of Simulated Lambda DNA Contours at Entanglement with Excluded Volume Effect

We generated simulated Lambda DNA contours at entanglement by constructing a 3D volumetric matrix of 2,000 × 1,000 × 200 pixels. Each cubic voxel within this matrix has a size of 5 (nm), approximately equal to the effective width of the excluded volume. This also translates to our imaging field size of 10 μm × 5 μm × 1 μm. Random seeds were generated, and the DNA segments were iteratively constructed while considering the excluded volume, utilizing the self-avoiding wormlike chain (SAWLC) model mentioned earlier (Figure 1E,F). These calculations were implemented in a custom MATLAB script and executed on the KAUST “Shaheen II” supercomputer. The results of our simulated Lambda DNA contour generation were exported as a 3D cell array containing the 3D coordinates of the generated contours for further processing.

### Deep Learning-Based Training for Predicting DNA Contours from Sparse Super-Resolution Microscopy Localizations

We utilized ANNA-PALM, a computational strategy that uses artificial neural networks for reconstructing super-resolution views from sparsely acquired localization images.^6^ Our training process involved using the simulated super-resolution localizations that we generated for ten thousand Lambda DNA molecules at entanglement, which were exported as CSV files and then imported into the training algorithm following the guidelines provided in the training documentation (https://github.com/imodpasteur/ANNA-PALM). The trained model’s output was exported as an executable JAR file compatible with ImageJ for predicting the DNA contours from 8-bit images generated from the sparse localization data. We note that the successful training of the model required CUDA version 10 and cuDNN version 7.6.5, as other versions resulted in errors during the training process. Additionally, to ensure successful training, we followed a specific procedure where ANNA-PALM’s example CSV files were first opened in the Origin Pro 2019 software. The header from these files was then copied and pasted into a new workspace. Subsequently, simulated or experimental data was appended below this new header, and the entire data set was exported as a CSV ASCII file. This method was crucial for running the training on custom CSV files without encountering errors. We utilized the Structural Similarity Index (SSIM) to quantitatively assess the structural dissimilarity between the simulated and predicted images. The SSIM is a metric measuring the similarity between two images. A value approaching 1 indicates high similarity, whereas values significantly below 1 indicate dissimilarity between the images. In this analysis, the SSIM values serve as a measure to assess the validity and quality of our predicted images under different imaging conditions.

### Preparation of Cy5-Labeled ssDNA Samples for Single-Molecule Imaging

A 10 μL aliquot of Cy5-labeled ssDNA (1 nM) was deposited onto a clean coverslip. A second coverslip was then placed on top of the droplet to spread the sample and ensure uniformly adsorbed ssDNA molecules on the glass surface. The ssDNA was incubated at room temperature for 10 min to allow adsorption. Then, the coverslips were carefully separated and washed to remove excess ssDNA, and then the coverslips were air-dried briefly before the deposition of the glycerol-switching buffer mixture. A glycerol solution (80%) was prepared by diluting glycerol with a concentrated solution of switching buffer to achieve the desired viscosity while maintaining the optimal concentrations of switching buffer components (see Sample Preparation). To image the sample, a double-sided adhesive tape was used to create a well on the coverslip surface, providing a confined area for the deposition of the glycerol-switching buffer mixture. This ensured that the Cy5-ssDNA labeled surface was covered with a sufficient volume of the imaging solution. The switching buffer was mixed with glycerol directly before imaging, and vigorous mixing was necessary to ensure thorough dispersion of the switching buffer throughout the glycerol solution.

### Detection and Tracking of Bright and Dark Segments of Predicted DNA Contours

We employed a multistep image processing approach to identify the bright DNA contours and the dark segments. First, we applied image thresholding to convert the grayscale images into binary images, segmenting the regions of interest (ROIs). The boundaries of the ROIs (detected using the marching squares algorithm (from the scikit-image Python library)) were highlighted using blue contours, as shown in Figure 6A-C. Subsequently, we quantified the distance between adjacent DNA contours by calculating the minimum distance between corresponding points on neighboring contours. This process involved computing pairwise distances between points along the contours and identifying the minimum distance as the dark segment size. The centers of these dark segments were then calculated. We implemented a tailored methodology to assess the dynamics of the dark segments within the DNA contours. To ensure consistent alignment for subsequent tracking, we preprocessed the data by analyzing linear molecules and rotating them to be parallel to the horizontal axis (Figure S25). This step facilitated the tracking of dark segment centers as one-dimensional motion. The tracking process connected these centers across consecutive frames through an iterative procedure. The iterative process started at the frame displaying the highest count of centers for dark segments, and connecting these centers across successive frames. Specifically, we identified the nearest centers in subsequent frames with a distance threshold of 300 nm (Supporting Note 4 and Figure S26), linking them both temporally (connecting to the nearest center in the next frame) and spatially (connecting to the nearest center in any of the subsequent frames if it resulted in a shorter distance). After establishing these connections, we selected the shortest tracks containing the largest number of centers. This methodological approach enabled accurate and efficient tracking of the dynamic centers within the analyzed data.

We note that dark segments are defined as regions extending beyond 300 nm that show a very low localization probability throughout the imaging period. These segments predominantly showed a complete absence of localization across frames, which we used to generate the tracks (Figure S23). They occasionally exhibited sparse blinking events. During these rare events, typically occurring in a limited number of frames, ANNA-PALM reconstructs the continuous contour (Figure S21), allowing the calculation of fluorescence on time in these regions (Figure S17B).

### Determination of the Viscosity of the Samples

To determine the glycerol concentration that matched the viscosity of the DNA matrix, we tracked 50 nm beads within the entangled DNA matrix. We found a measured diffusion coefficient of 0.056 μm^2^ s^–1^, corresponding to a viscosity of 78 mPa s, which corresponds to 80% glycerol (v/v) according to known glycerin solution viscosity tables.^35^ Single-molecule tracking of 50 nm fluorescent beads in 80% glycerol provided viscosity measurements of 80 cP, confirming our results.

## Supporting Information Available

The simulation of self-avoiding DNA contours, and calculations of labeling density, average number of localizations per emitter, and volume fraction of lambda DNA, Supporting Notes describing the localization density required for the complete contour reconstruction, single-molecule fluorescence characterization of Cy5 molecules under different environmental conditions, effect of DNA sequence and structure on fluorescence of covalently attached Cy5 dye, the rational for the 300 nm threshold in the generation of the tracks of the dark segments, optical artifacts in fluorescence intensity variations, and assignment of the observed dark segments to entanglement loci, Probability distribution of bending angles in three dimensions as a function of DNA segment length (Figure S1), examples of simulated self-avoiding DNA contours at entanglement (Figure S2), frequency distribution of the standard deviations of single molecule localization on Cy5 molecules conjugated to Lambda DNA (Figure S3), simulated DNA contours and localization with random noise (Figure S4), simulated 8-bit images of DNA contours used for training ANNA-PALM (Figure S5), simulated contours of Lambda DNA generated with varied signal-to-noise ratios (Figure S6), effect of random noise on the performance of ANNA-PALM prediction (Figures S7–S9), effect of localization precision and random noise on the performance of ANNA-PALM (Figure S10), experimentally obtained SMLM images of Cy5-labeled DNA (Figure S11), temporal resolution assessment of DNA contour reconstruction (Figure S12), illustrations of DNA contour reconstruction at different temporal resolutions (Figure S13), experimentally obtained SMLM image of a Cy5-labeled Lambda DNA deposited on a glass surface in the switching buffer (Figure S14), experimentally obtained SMLM image of a Cy5-labeled DNA deposited on a glass surface in the switching buffer with the matrix DNA (Figure S15), effect of the diffusion barrier on the frequency distributions of the fluorescence on-time of Cy5 conjugated to ssDNA (Figure S16), frequency distributions of the fluorescence on-time of Cy5 conjugated to Lambda DNA in bright and dark segments (Figure S17), examples of lambda DNA contours and localization patterns revealing bright and dark segments (Figure S18), predicted DNA contours and dark segment centers of Lambda DNA suspended in homogeneous solution and in entangled DNA matrix (Figures S19–S21), examples of the temporal dynamics of dark segments’ centers of a predicted contours from Lambda DNA in DNA matrix (Figure S22), examples of the time-lapse images of the predicted contours obtained from Lambda DNA in a DNA matrix (Figure S23), examples of the temporal dynamics of dark segments’ centers of a predicted contours from Lambda DNA in 80% glycerol (Figure S24), center positions of the dark segments predicted at different time intervals (Figure S25), comparison of dark segment sizes in original and ANNA-PALM predicted images (Figure S26), averaged mean square displacement plot versus time lag obtained from the time trajectories of the center positions of the dark segments detected for Lambda DNA suspended in 80% glycerol (Figure S27), additional references (PDF)
