# Supplementary material for: Deep Learning and Single-Molecule Localization Microscopy Reveal Nanoscopic Dynamics of DNA Entanglement Loci
Source: ACS Nano. 2025 Feb 4;19(6):6236–49. doi: 10.1021/acsnano.4c15364 (PMC11841032; doi:10.1021/acsnano.4c15364)
Supplement: Supplementary file 1 — nn4c15364_si_001.pdf [file nn4c15364_si_001.pdf]

Supporting Information

Deep Learning and Single-Molecule Localization Microscopy Reveal Nanoscopic Dynamics of DNA Entanglement Loci

Maged F. Serag, Maram Abadi, Hajar Al-Zarah, Omar Ibrahim and Satoshi Habuchi**

Biological and Environmental Science and Engineering Division, King Abdullah University of Science and Technology, Thuwal 23955-6900, Saudi Arabia

Contents

Supporting Methods

Supporting Notes 1 – 6

Supporting Figures 1 – 27

References

Supporting Methods

Simulating Self-Avoiding DNA Contours

A three-dimensional simulated polymer can be defined by the coordinates of its joints $\{S_0, S_1, S_2, \ldots, S_{n-1}\}$, where n is the total number of joints in a polymer of $n-1$ segments. The segments will be denoted by the vectors $\{\vec{V}_1, \vec{V}_2, \vec{V}_3, \ldots, \vec{V}_{n-1}\}$.

Given the previous segment $\vec{V}_{r-1}$, the coordinates of the last joint S_{r-1}, the segment length l, and the bending angle α, the next segment $\vec{V}_r$ can be calculated. This information alone allows infinitely many points to form a circle in 3D space. A random point is selected at a random angle β between a random vector $\vec{V}_R$ parallel to the plane of the circle. The component of $\vec{V}_r$ parallel to the circle, $\vec{V}_{r,p}$, is found by solving equations (S1 –S3):

$$\vec{V}_{r,p} \cdot \vec{V}_{r-1} = 0 \tag{S1}$$

$$\left|\vec{V}_{r,p}\right|^2 = l^2 \sin^2 \alpha, \qquad 0 \le \alpha \le 180 \tag{S2}$$

$$\vec{V}_{r,p} \cdot \vec{V}_R = l \sin \alpha \left|\vec{V}_R\right| \cos \beta, \quad 0 \le \beta < 360 \tag{S3}$$

After solving for $\vec{V}_{r,p}$, $\vec{V}_r$ and S_r are found:

$$\vec{V}_r = \vec{V}_{r,p} + (l \cos \alpha) \frac{\vec{V}_{r-1}}{|\vec{V}_{r-1}|} \tag{S4}$$

$$\vec{V}_r = \vec{V}_{r,p} + (l \cos \alpha) \frac{\vec{V}_{r-1}}{|\vec{V}_{r-1}|} \tag{S5}$$

The next goal is to ensure that a sphere of diameter ω centered at S_r does not intersect with the spheres surrounding the nearby joints of the polymer. In other words, we want to find a point S_r such that it is a distance l from S_{r-1} and is a minimum distance of ω from the neighboring points. To do so, we consider all the possible coordinates for S_r only when $\vec{V}_r$'s length is fixed to l (mean segment length). This set of coordinates that we shall call M forms a sphere with radius l. Consider

the simplified situation where, besides the point S_{r-2}, there is only one point p_1 a distance less than $l + \omega$ from S_{r-1} (i.e., the maximum distance a point can be from S_{r-1} to restrict the possible coordinates S_r). The existence of the point p_1 in the neighborhood of S_{r-1} restricts the set of possible points to M_1, a sphere cap. Adding another point p_2, we consider only its imposed restriction, resulting in another set of allowed points, M_2. The set of allowed points with the existence of both p_1 and p_2 is given by the intersection of M_1 and M_2:

$$M_1 \cap M_2 \quad \text{(S6)}$$

Thus, the set of allowed points M_a with the existence of m points is:

$$M_a = M_1 \cap M_2 \cap M_3 \cap \ldots \cap M_m \quad \text{(S7)}$$

where M_i is the set of allowed points restricted by the existence of only p_i.

If $M_a = \emptyset$, there exists no direction that allows the polymer to avoid itself, then a number of previous points s are deleted, and the process starts from S_{r-s-1}.

Calculations of labeling density and average number of localizations per emitter

- The molecular weight of Lambda DNA is 31.5 × 10^6 Daltons. 31.5 × 10^6 gm/mole = 31.5 × 10^9 mg/mole
- The concentration used is 10 mg/ml. 10 mg is 10 ÷ 31.5 × 109 = 3.1746 × 10^{-10} moles
- Number of molecules is 3.1746 × 10^{-10} × 6.0221409 × 10^{23} = 1.9118 × 10^{14} molecules in 1 ml
- Number of molecules in cubic micrometers is 1.9118 × 10^{14} ÷ 1 × 10^{12} = 191 molecules in μm^3
- Number of molecules in imaging field 5 µm × 10 µm × 1 µm micrometers is 50 × 191 = 9,559 molecules
- Number of labeled molecules in imaging field 5 µm × 10 µm × 1 µm micrometers is 9,559 ÷ 850 = 11.2 molecules (0.22 molecules in 1 cubic micrometers)

- Labeling density is one dye per 5 – 15 bp; this means we have
- 48,502 ÷ 5 = 9,700 dye molecules per molecule of DNA
- 48,502 ÷ 15 = 3,233 dye molecules per molecule of DNA
- Total dye molecules in the imaging field are 3,233 × (9,559/850) = 35,563 – 106,700 dye molecules
- We have 300,000 total localizations; this means we have, on average, 2.8 – 8.4 localization of each dye (about five localizations per label)

Calculations of the volume fraction of Lambda DNA

Density of DNA = 1.7 gm cm^{-3}[1] = 1,700 mg cm^{-3}

Volume of 10 mg = 10 ÷ 1,700 = 0.0059 cm^3

Volume fraction = 0.0059 cm^3 ÷ 1 cm^3 (1ml) = 0.0059

Supporting Notes

1. **Localization density required for the complete contour reconstruction**

It is important to distinguish between our noise sensitivity analysis and actual experimental conditions. The criteria we established (3 localizations per fluorophore, 10% maximum noise, etc.) represent our algorithm's performance limits under noisy conditions, as seen in experimental scenarios like Figure 2D and Figure S15. However, our actual analysis follows the protocol established in our previous work,(2) where we isolate molecules and minimize background noise. Under these low-noise experimental conditions, ANNA-PALM successfully reconstructs molecular contours even from sparse localizations. Figure S8B demonstrates that with minimal

noise (< 3%) and just one localization per fluorophore, we achieve SSIM values above 0.9, indicating highly accurate predictions.

The localization density required for the complete contour reconstruction in our imaging conditions (one localization per fluorophore) is approximately 200 – 600 dye molecules per micrometer of DNA, based on our labeling density of 5 – 15 bp per dye molecule. As detailed in the Supporting Methods, our imaging captured 300,000 total localizations during about 60 s. For a Lambda DNA molecule with an observed maximum contour length of 13 μm (Figure S12) in our experiments, we achieved roughly 230 localizations per micrometer in a 0.5 s interval. This density is sufficient for the complete contour reconstruction during early imaging time points (Figure S12A). Photobleaching gradually reduces this number toward the end of imaging time, especially after 20 seconds, and therefore, a complete contour cannot be generated. When extending the sampling interval to 2 s, we increased the total localizations to approximately 920 per micrometer (4 × 230), ensuring reliable contour reconstruction even at later time points.

In conclusion, while increasing integration time improves reconstruction completeness through additional localizations, our method reliably reconstructs contours even at sub-second temporal resolution under proper experimental conditions.

2. Single-molecule fluorescence characterization of Cy5 molecules under different environmental conditions

To characterize the kinetics of Cy5 fluorescence at different viscosities, we recorded single-molecule fluorescence trajectories of ssDNA-Cy5 molecules deposited on glass surfaces in various environment settings and at a video rate of 33 Hz. We processed the fluorescence traces using a zero-phase filter to remove noise while preserving signal amplitudes and shapes. We determined on-time values by fitting these bursts with a Gaussian function, using their FWHM when the

Gaussian r-squared value exceeded 0.5. When the r-squared value was below 0.5, on-time was determined by simply measuring burst widths. Signals with r-squared values less than zero and those that couldn't be optimized within 1,000 iterations were disregarded. All experimental conditions were treated consistently in terms of sample preparation, concentration, laser power, and imaging conditions. In addition, signal processing involved setting the filter order to 2 and the cut-off parameter to 0.1, while scaling signal amplitudes between 0 and 10 (a.u.) for convenience in fitting parameter initialization and threshold setting to identify peak heights before the Gaussian fitting.

3. Effect of DNA sequence and structure on fluorescence of covalently attached Cy5 dye

The fluorescence emission of Cy5 labels exhibits a shorter on-time when covalently attached to a double-stranded DNA (dsDNA) compared to their attachment to single-stranded DNA (Figures. 5B, E). This difference arises from two key factors: the spatial separation of dye molecules and their proximity to the phosphate backbone. In ssDNA, the Cy5 labels are more spatially separated, whereas in dsDNA, the dye molecules are positioned closer to the negatively charged phosphate backbone. Consequently, the excited state lifetime and quantum yield of Cy5 are reduced in dsDNA, as evidenced by the observation that the fluorescence quantum yield of Cy3, a spectrally similar cyanine dye, decreases by a factor of 2.4 when transitioning from Cy3-labeled 5' ssDNA to Cy3-labeled 5' dsDNA.[(3)]

As per the manufacturer, the Cy5 labeling of Lambda DNA proceeds via covalent conjugation of the dye to any reactive heteroatom within nucleic acids. The reactive group is linked to the Cy5 dye via a positively charged linker, which electrostatically interacts with the negatively charged phosphate backbone. This labeling scheme suggests that the conjugated Cy5 dyes do not directly

interact with the DNA bases (e.g., through π-π stacking). Consequently, the influence of DNA sequence on the fluorescence characteristics of the Cy5 dyes is expected to be minimal or negligible. Therefore, the observed variations in switching kinetics are more likely attributed to local environmental factors rather than sequence-specific interactions.

4. The rationale for the 300 nm threshold in the generation of the tracks of the dark segments

We found that a threshold of 300 nm represents the upper limit for the size of dark segments that ANNA-PALM effectively fills. Dark segments smaller than 300 nm are reconstructed by the algorithm, resulting in a continuous predicted DNA contour. However, when dark segments exceed 300 nm, the algorithm's ability to predict the contour diminishes, leading to discontinuities in the predicted contour (Figure S26). If the distance between two centers in consecutive frames is less than 300 nm, it is highly likely that they originate from the same dark segment, as this distance is within the size range of the lower limit of a single dark segment identified by ANNA-PALM. In this case, connecting these centers allows for the accurate tracking of the dark segment's position over time. However, if the distance between two centers in consecutive frames exceeds 300 nm, it suggests that they may belong to different dark segments. This is because 300 nm represents the upper limit for the size of a single dark segment identified by ANNA-PALM. When centers are separated by a distance greater than the size of the lower limit of a single dark segment, it becomes more probable that they originate from distinct dark segments rather than the same one.

When a dark segment contains a localization point(s) that effectively divides it into smaller sub-segments, the algorithm can bridge these gaps and connect the contour if the sub-segments are sufficiently small. However, when the dark segment is larger and contains fewer localization points, the algorithm may predict a combination of short bright segments and dark segments,

depending on the spacing between the localization points and the segment boundaries. Therefore, by setting a constant threshold of 300 nm, the tracking algorithm ensures that connected centers belong to the same dark segment, and preventing artifacts. This standardized threshold enables consistent comparison of dark segment dynamics between Lambda DNA in a matrix and 80% glycerol solution. The presence of multiple connectable centers in individual frames forming a network of tracks indicates the random distribution of localizations and the centers, distinguishing them from persistent structures with single trackable centers across frames (see Figures. S22 – S24). This threshold approach facilitated the distinction between Lambda DNA in both matrix and 80% glycerol.

5. Ruling out optical artifacts in fluorescence intensity variations

It's important to note that our experimental setup minimizes the potential influence of sample depth and unlabeled DNA on the observed fluorescence intensity variations. Due to the low concentration of fluorophores in the "on" state at any given time, light absorption by surrounding molecules, even those located at different heights within the sample, is unlikely to significantly affect the fluorescence intensity or switching behavior of the target molecules. Furthermore, the sparse labeling and stochastic switching nature of our approach renders the potential for scattering by unlabeled DNA negligible. The low concentration of "on" state fluorophores makes it statistically unlikely for unlabeled DNA to consistently block or significantly scatter light from the labeled molecules within the observed timeframe. These considerations support our interpretation that the observed non-random distribution of localizations along the contours is primarily due to entanglement effects rather than optical artifacts or variations in sample depth.

6. The assignment of the observed dark segments to entanglement loci

When ANNA-PALM detects gaps larger than 300 nm, it cannot distinguish between the gaps originating from the dark segments and those originating from the stochastic nature of the switching events. Therefore, we compared the gaps detected for the entangled DNA and reference DNA in the homogeneous solutions (Figure 6). Our analysis shows much higher gap occupancy for the entangled DNA, which strongly suggests that the majority of the gaps detected are associated with dark segments. This discussion is further strengthened by the comparison of the MSD versus time lag plots obtained for the entangled DNA and reference DNA in the homogeneous solutions. The former shows a sub-diffusional behavior (Figure 7C). On the contrary, the latter exhibits no correlation between the MSD and time lag (Figure S27), which suggests the absence of the correlation between the gap positions detected in the consecutive frames. Together with the very short trajectories of the dark segment dynamics (Figure S24), the result strongly suggests that the dark segment dynamics observed for the entangled DNA are mainly associated with the dynamics of entanglement loci, whereas the dark segment dynamics observed for the reference DNA in the homogeneous solutions is dominated by the stochastic nature of the switching events.

Supporting Figures

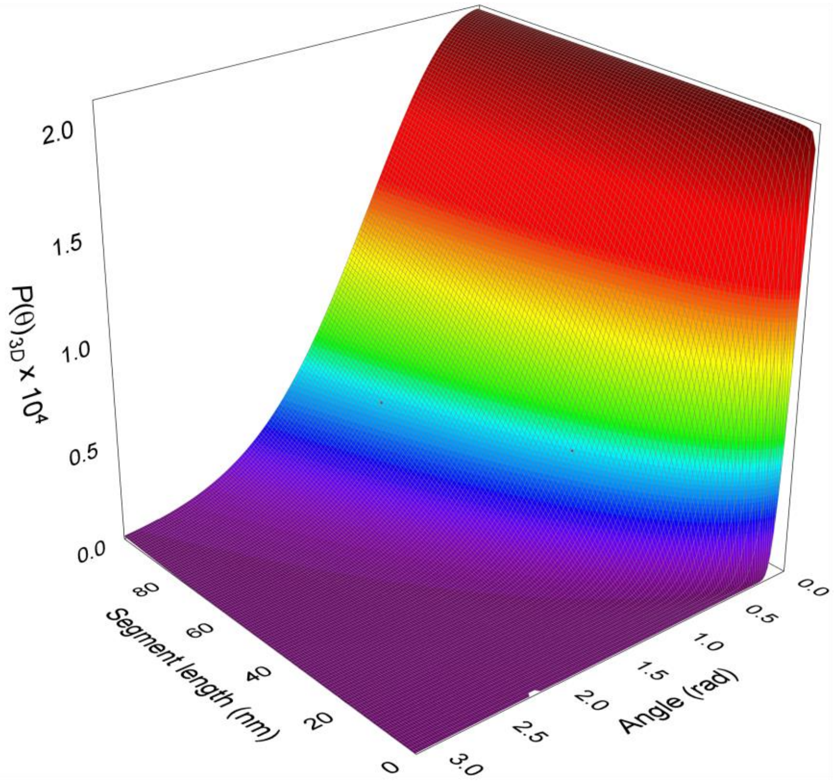

Figure S1. Probability distribution of bending angles $P(\theta)_{3D}$) in three dimensions as a function of DNA segment length.

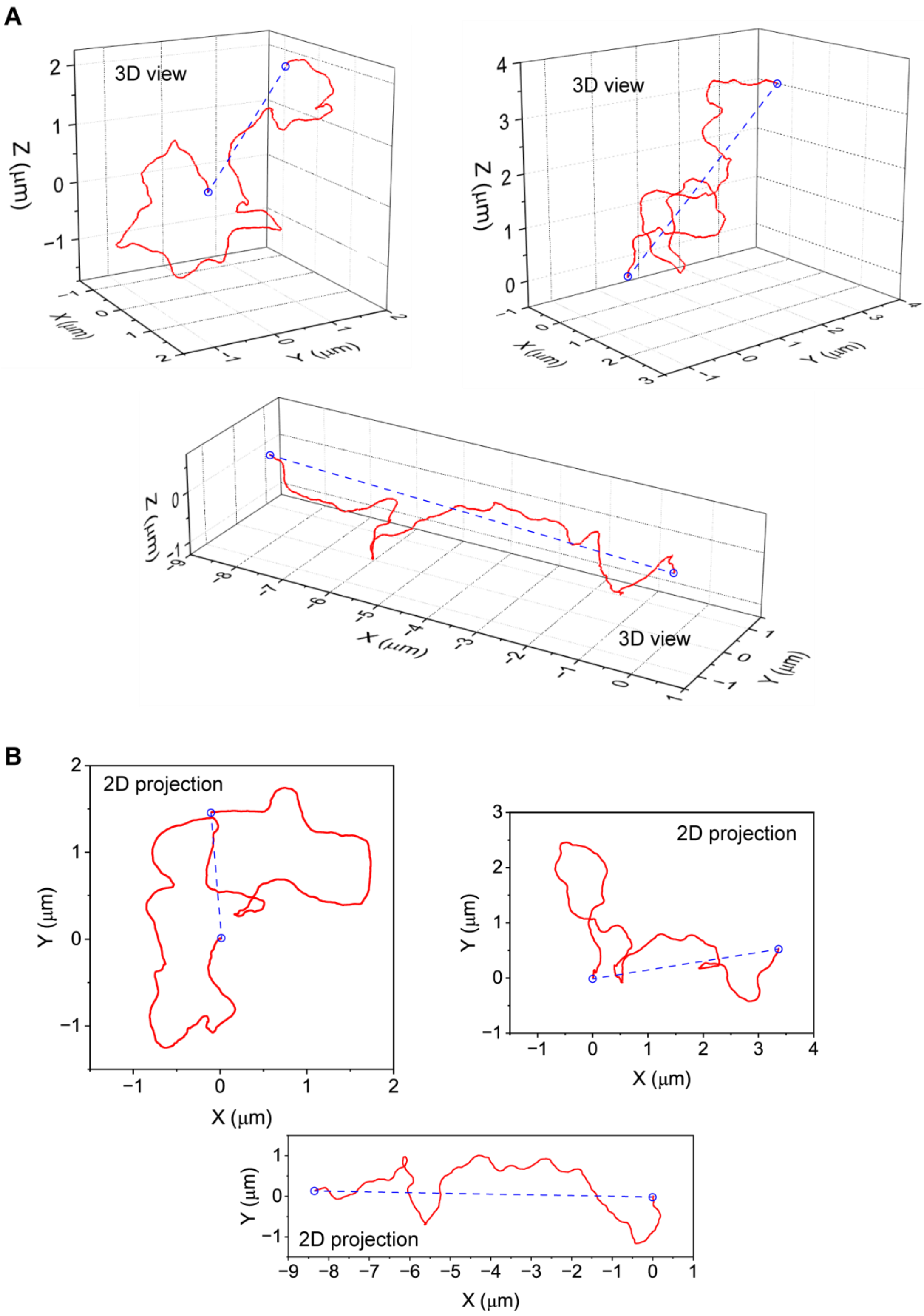

Figure S2. Three examples of simulated self-avoiding DNA contours at entanglement. (A) 3D view. (B) 2D projection. The dashed lines show the end-to-end distance.

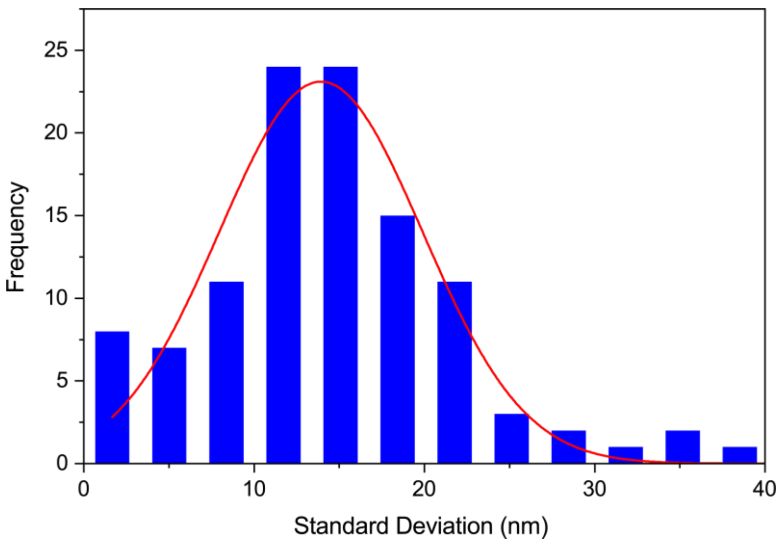

Figure S3. Frequency distribution of the standard deviations of single molecule localizations on Cy 5 molecules conjugated to Lambda DNA. The red line shows a Gaussian fitting.

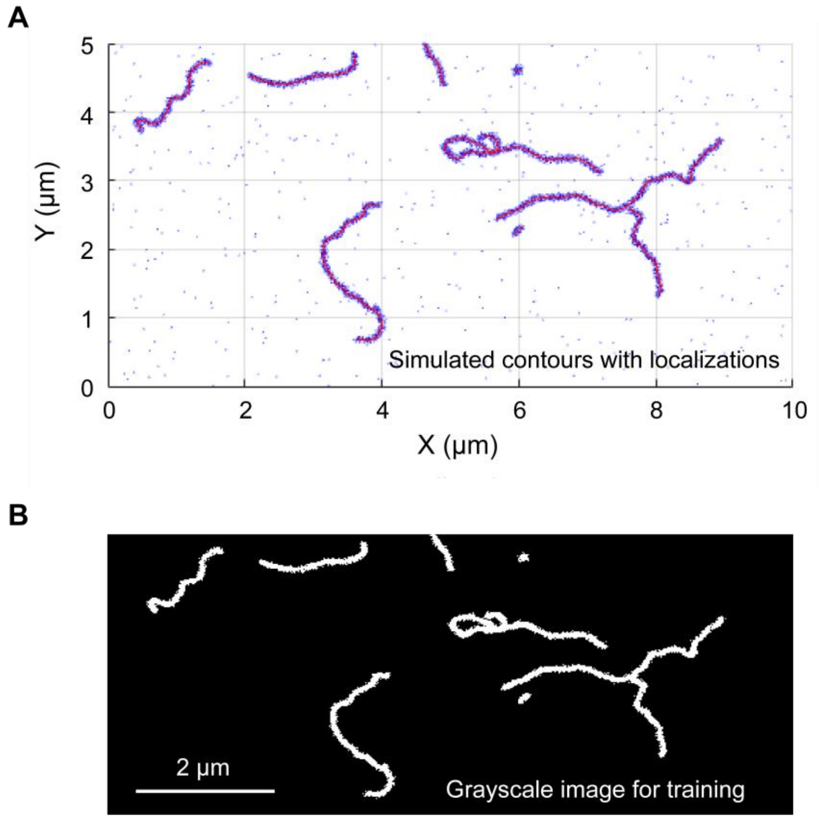

Figure S4. (A) Simulated DNA contours (red lines) and simulated localization with random noise (blue dots). (B) 8-bit grayscale image generated from the localizations shown in (A) used for training ANNA-PALM.

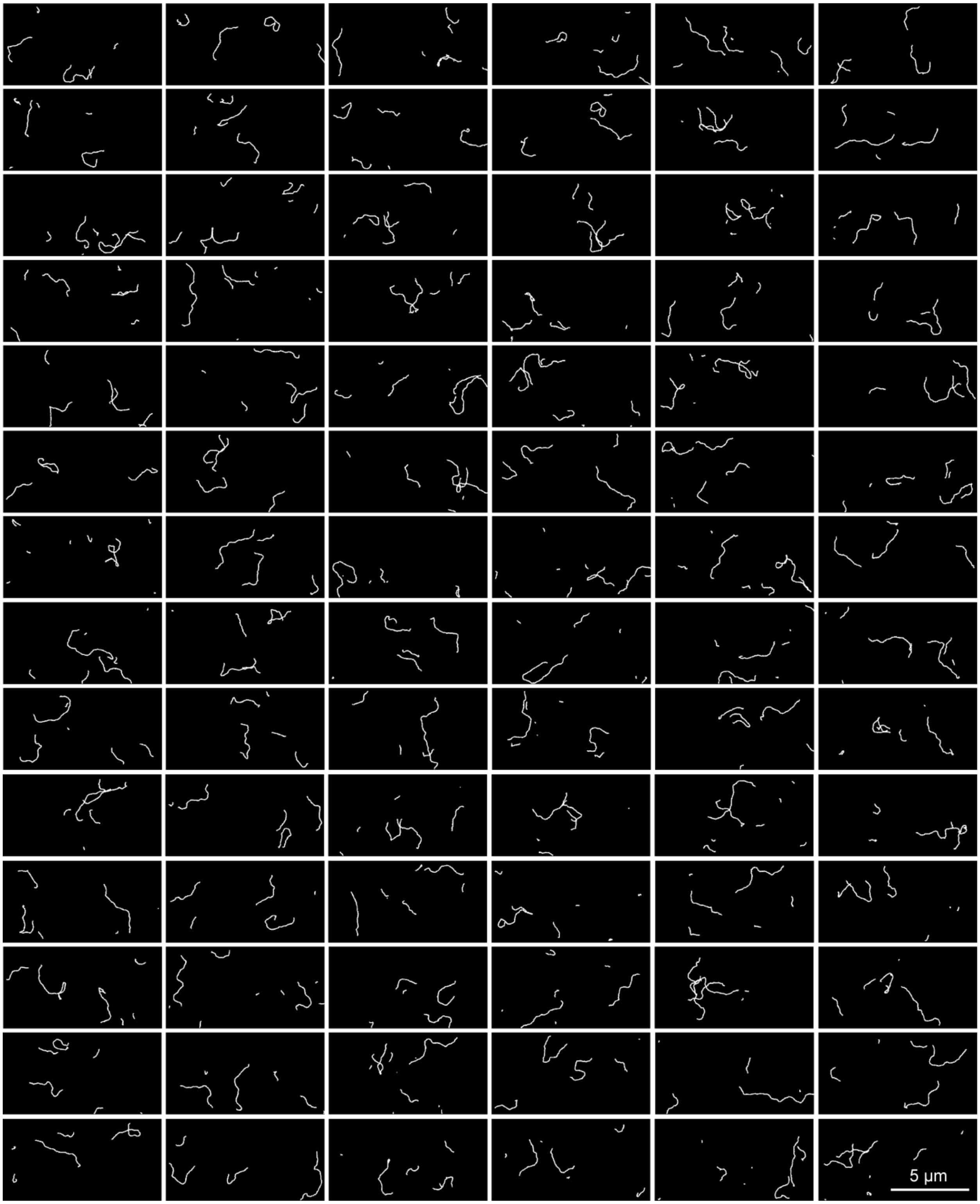

Figure S5. Simulated 8-bit images showing DNA contours used for training ANNA-PALM.

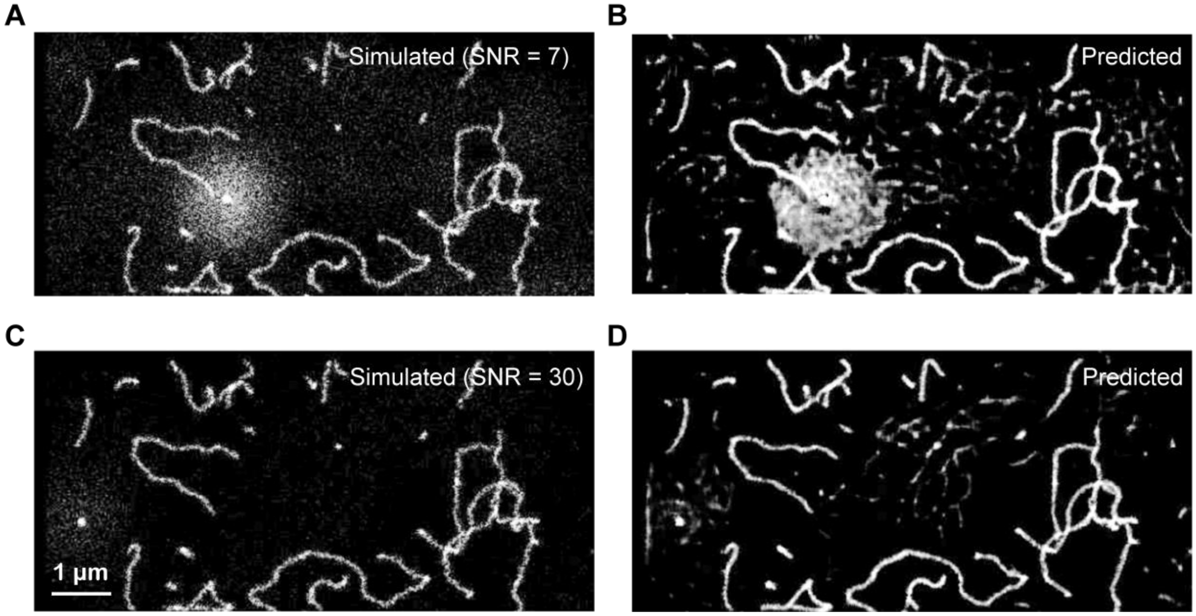

Figure S6. (A, C) Simulated contours of Lambda DNA generated with a signal-to-noise ratio (SNR) of (A) SNR = 7 and (C) SNR = 30. We incorporated regions characterized by dense localizations corresponding to defocused or densely labeled small DNA fragments within these simulated contours. (B, D) Predicted images of the DNA contours simulated in (A) and (C).

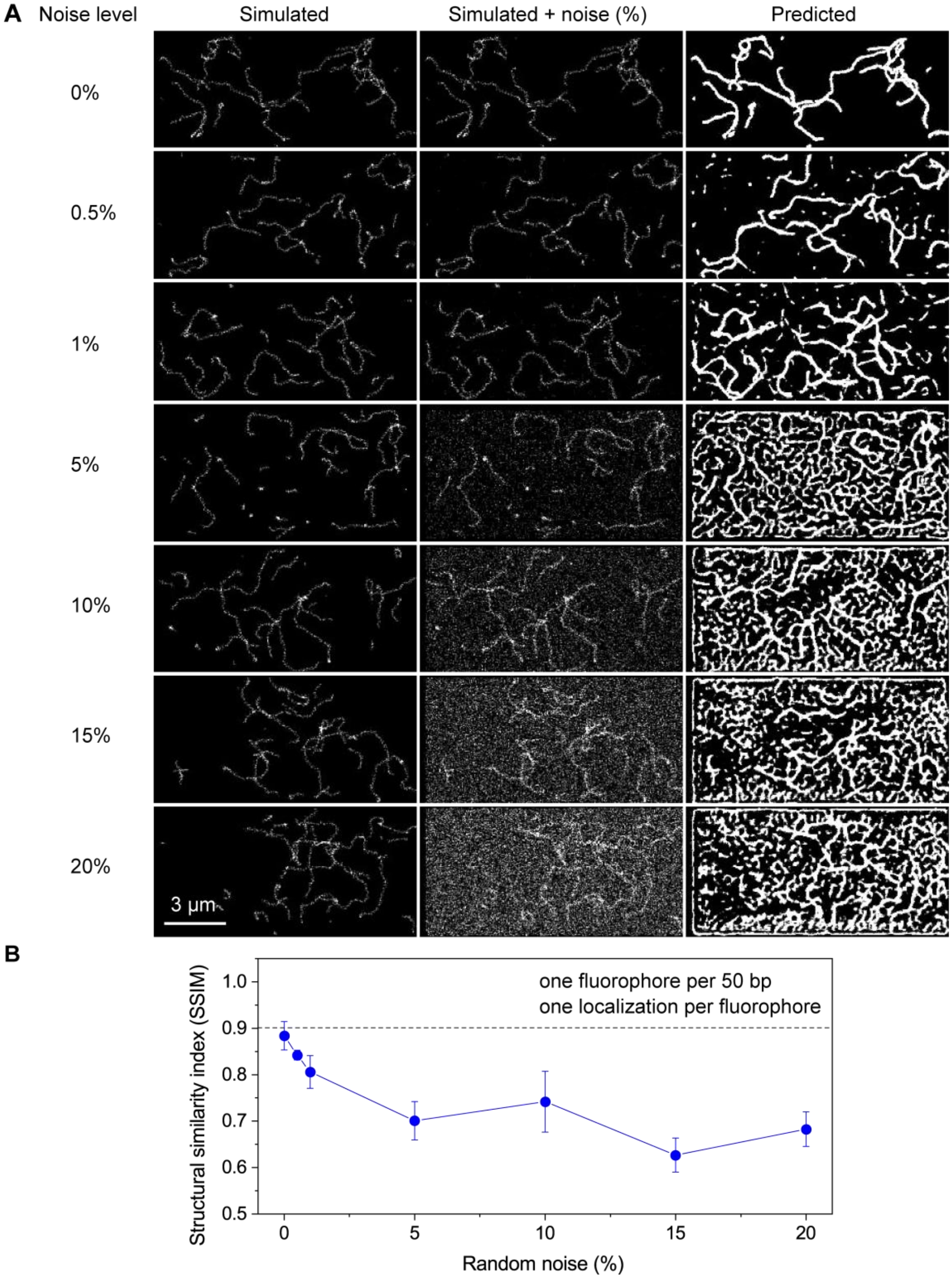

Figure S7. Effect of random noise on the performance of ANNA-PALM prediction. (A) Simulated contours of Lambda DNA at entangled conditions generated at the localization precision of 30 nm, labeling density of one fluorophore every 50 bp, and one localization per fluorophore (left), simulated contours with different noise densities (center), and the predicted images (right). (B) The structural similarity index (SSIM) obtained at different noise densities.

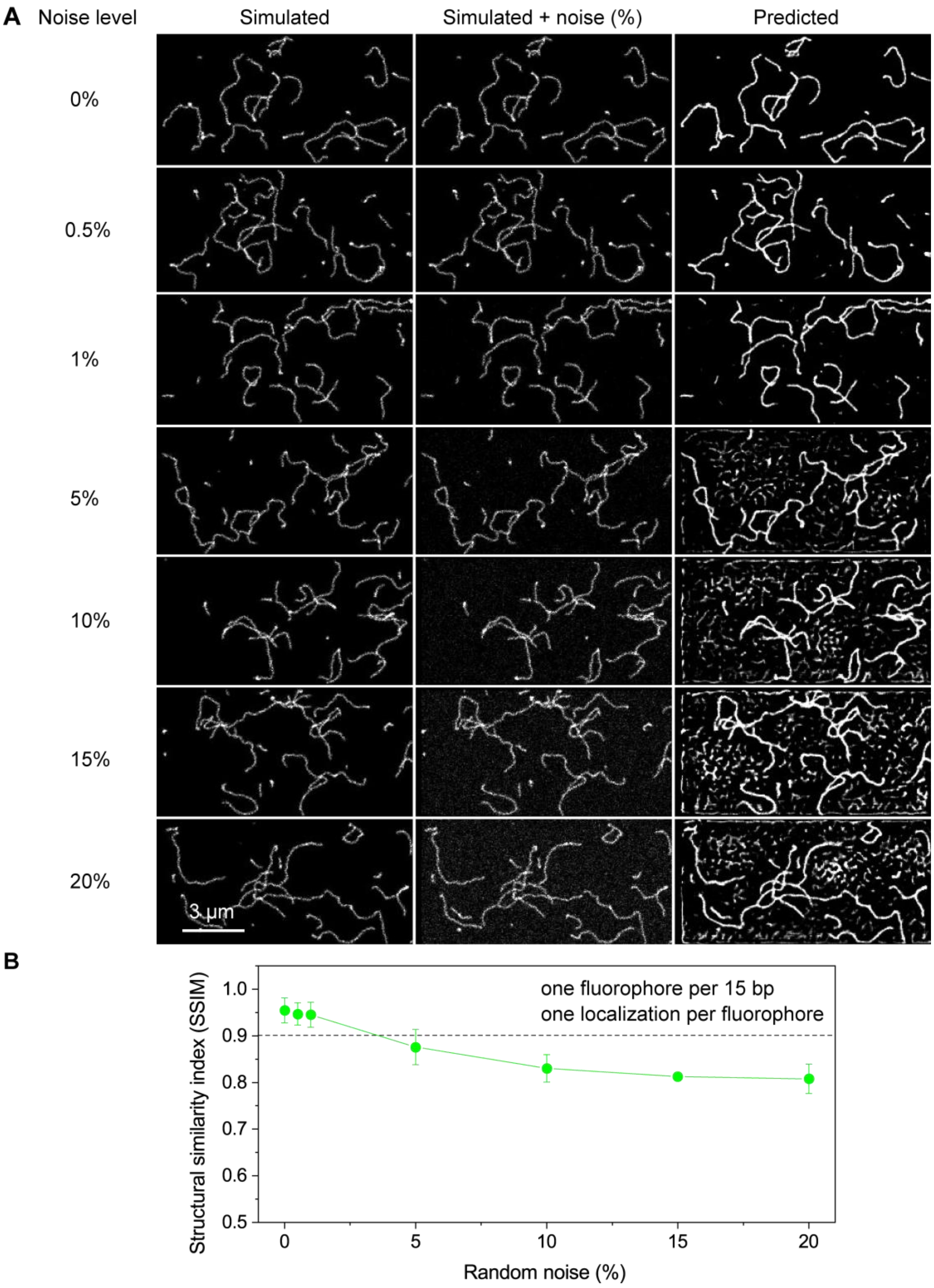

Figure S8. Effect of random noise on the performance of ANNA-PALM prediction. (A) Simulated contours of Lambda DNA at entangled conditions generated at the localization precision of 30 nm, labeling density of one fluorophore every 15 bp, and one localization per fluorophore (left), simulated contours with different noise densities (center), and the predicted images (right). (B) The structural similarity index (SSIM) obtained at different noise densities.

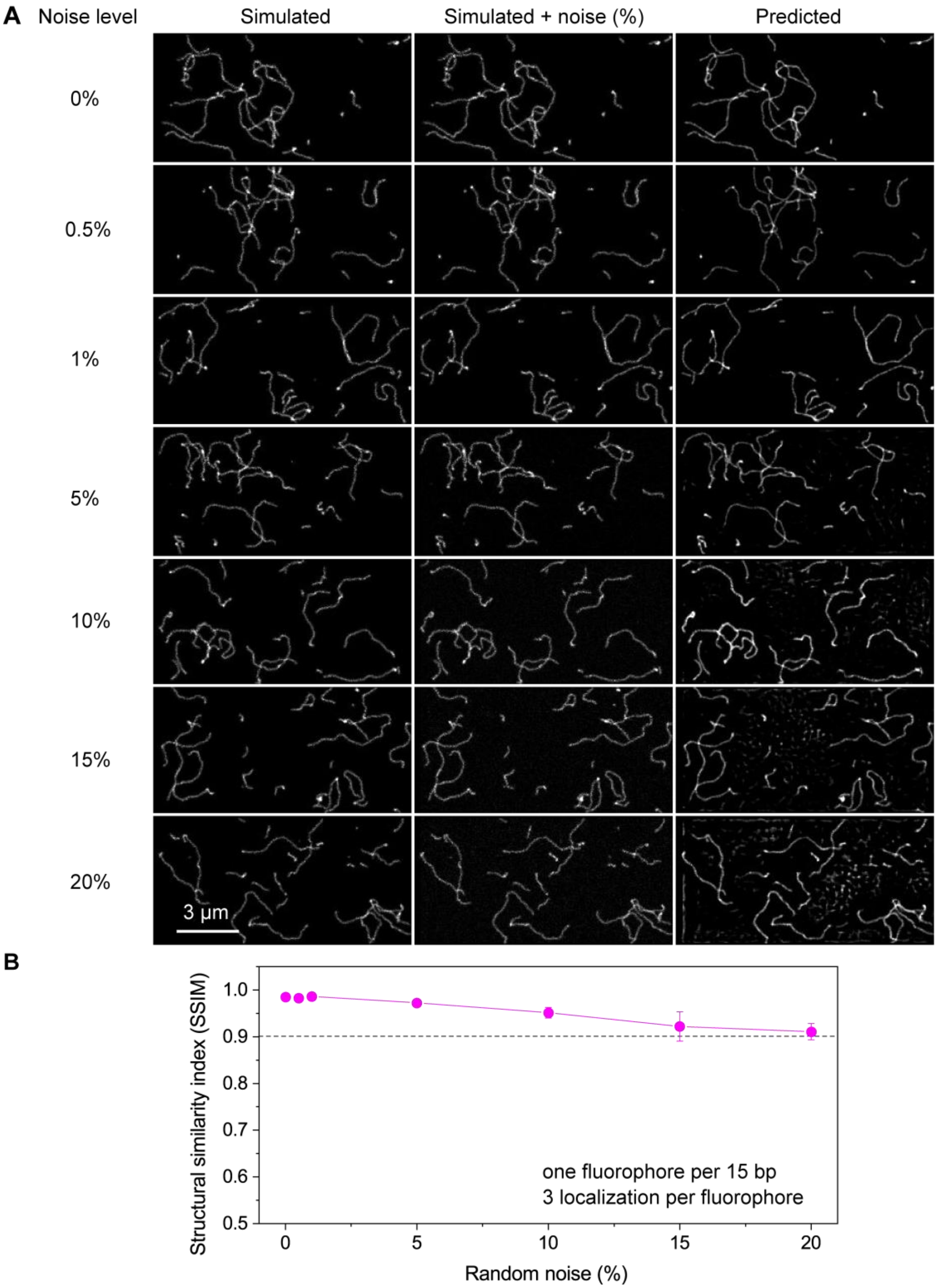

Figure S9. Effect of random noise on the performance of ANNA-PALM prediction. (A) Simulated contours of Lambda DNA at entangled conditions generated at the localization precision of 30 nm, labeling density of one fluorophore every 15 bp, and three localizations per fluorophore (left), simulated contours with different noise densities (center), and the predicted images (right). (B) The structural similarity index (SSIM) obtained at different noise densities.

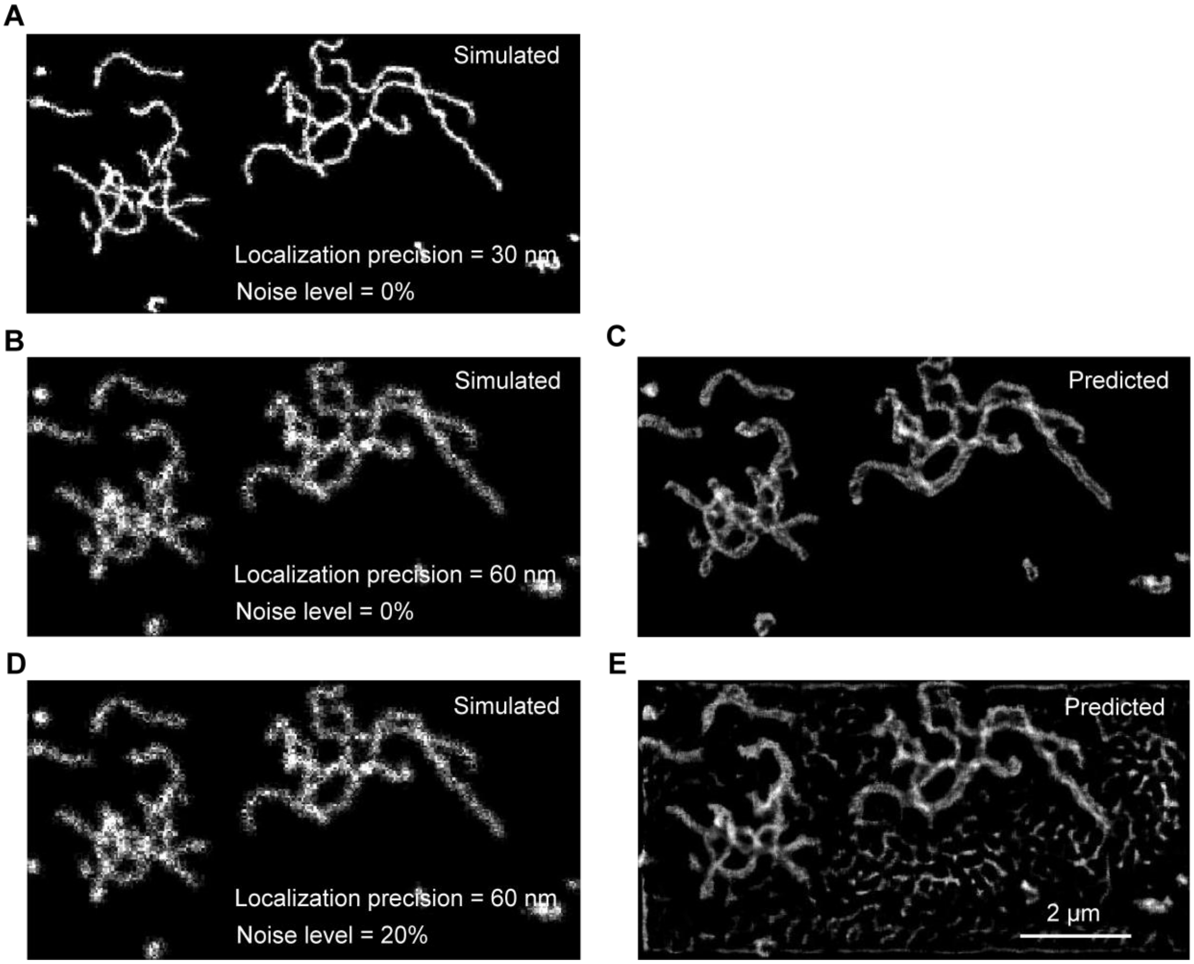

Figure S10. Effect of poor localization precision (SD = 60 nm) and 20% random noise on the performance of ANNA-PALM prediction. (A) Simulated DNA contours with a localization precision of 30 nm. (B, D) Simulated DNA contours with localization precision of 60 nm with (B) 0% noise and (D) 20% noise. (C, E) Predicted images of the DNA contours simulated in (B) and (D).

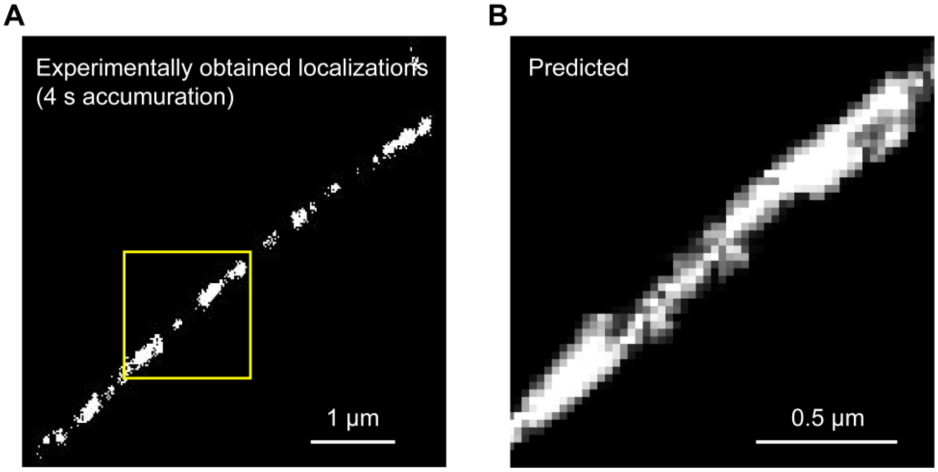

Figure S11. (A) Experimentally obtained SMLM image of Cy5-labeled Lambda DNA. The image was reconstructed using single-molecule images acquired during 4 seconds and converted to an 8-bit grayscale image. (B) Predicted image for the SMLM image in (A) (area highlighted by the yellow line) using ANNA-PALM.

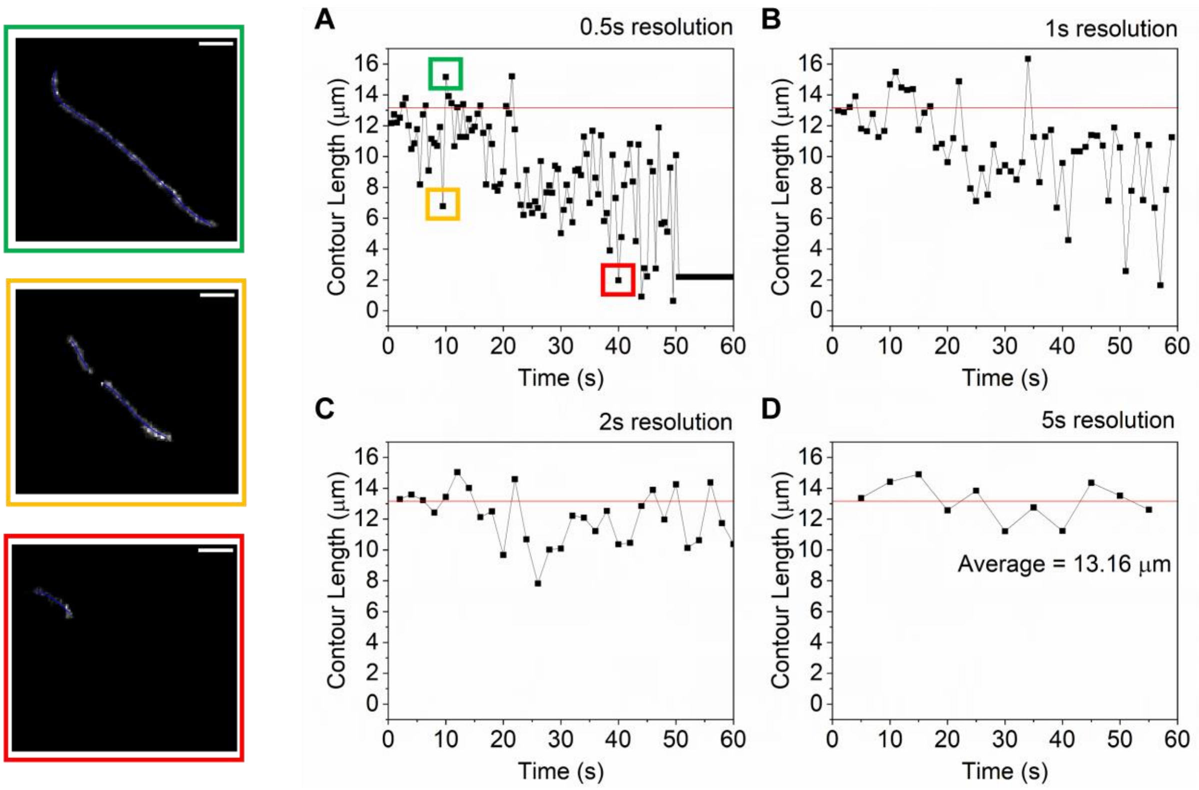

Figure S12: Temporal resolution assessment of DNA contour reconstruction. (A) DNA contour length fluctuations at 0.5s resolution. Each data point represents the length of a reconstructed contour from localizations gathered over 500 frames (0.5s at 1 kHz frame rate). The red line indicates the average molecule length (13.16 μm) calculated from contours generated at 5-second resolution (shown in (D)). (B–D) DNA contour length fluctuations at 1 s, 1.5 s, and 2 s resolutions, respectively. Left inset images: Color-coded examples of reconstructed contours corresponding to specific time points in (A), showing both complete and partially generated contours. Scale bar = 2 μm.

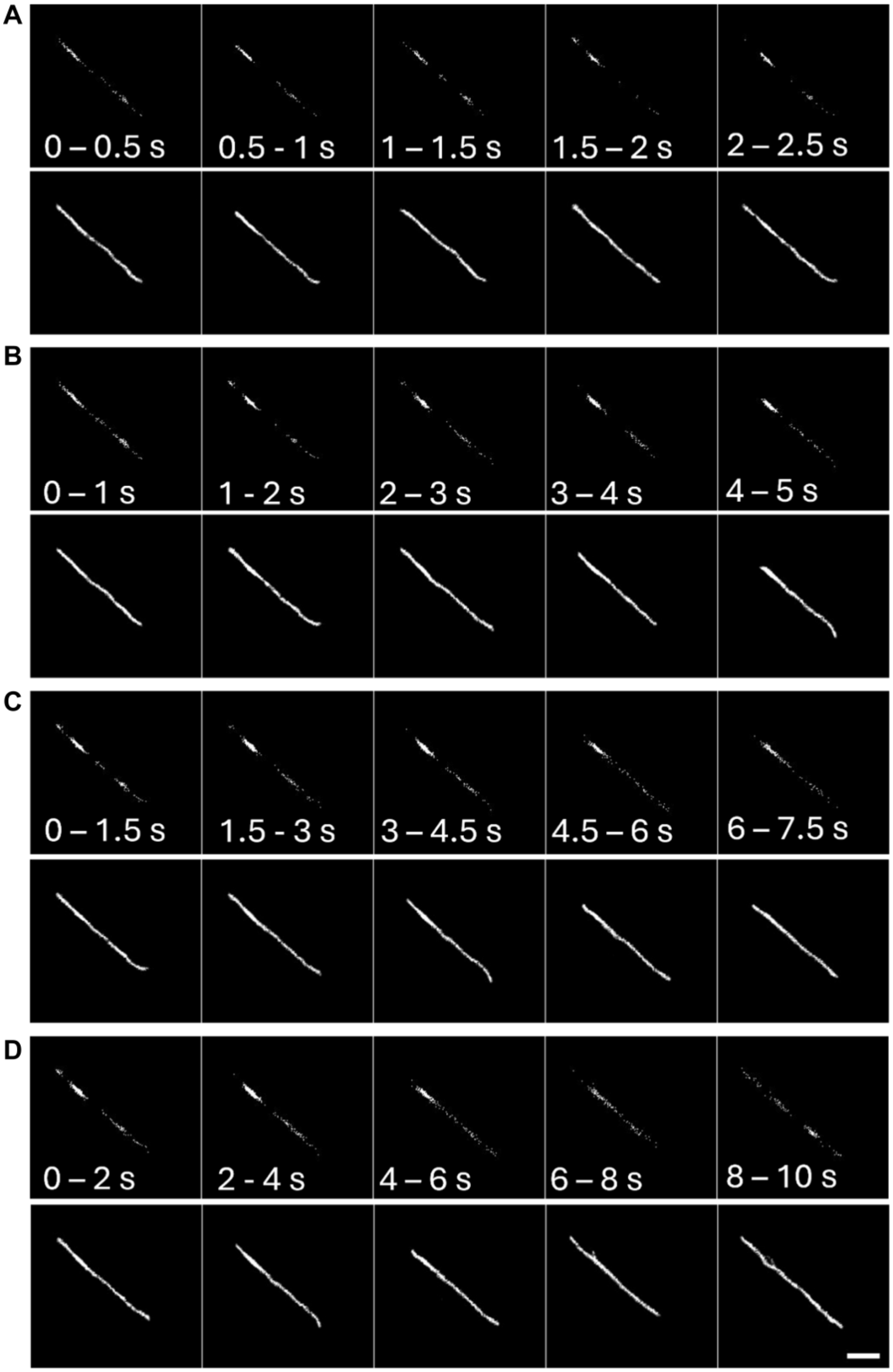

Figure S13: Illustrations of DNA contour reconstruction at different temporal resolutions. Each panel (A-D) shows an 8-bit grayscale image of raw localizations at various temporal resolutions: (A) 0.5 s, (B) 1 s, (C) 1.5 s, and (D) 2 s (top) and corresponding DNA contour reconstructed using the ANNA-PALM algorithm (bottom). Scale bar = 3 μm.

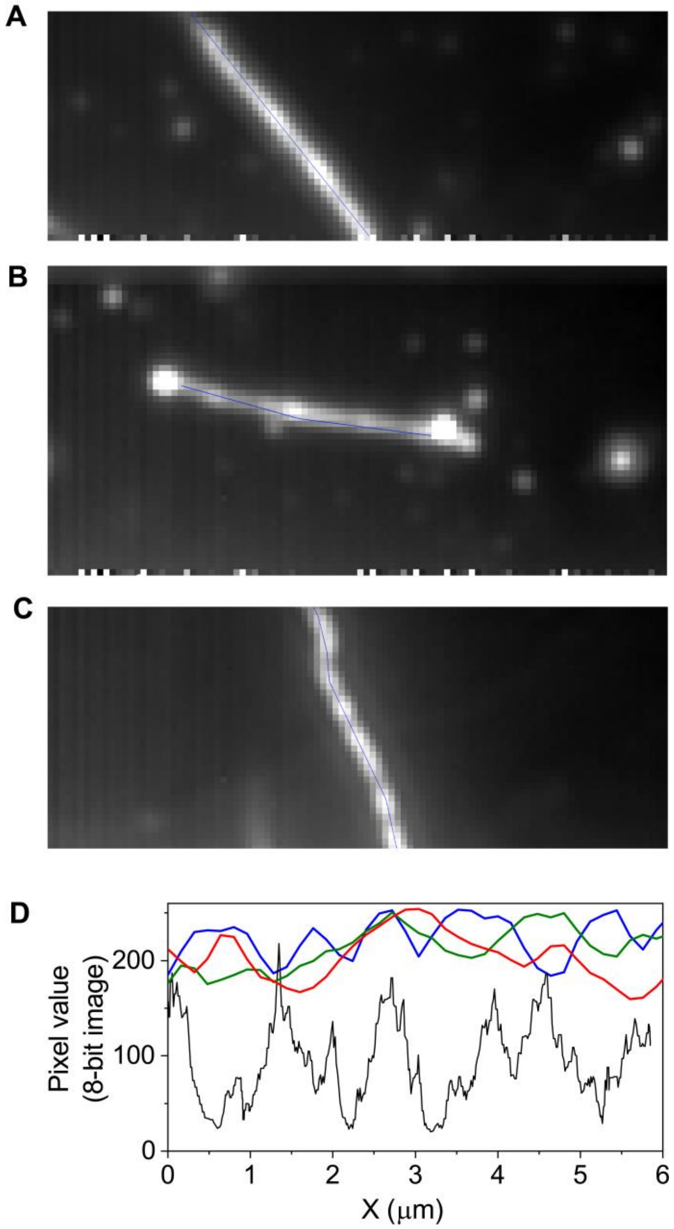

Figure S14. (A-C) Experimentally obtained SMLM image of a Cy5-labeled Lambda DNA deposited on a glass surface in the switching buffer. The image was reconstructed using single-molecule images acquired during 50 seconds and converted to an 8-bit grayscale image. The blue line represents the DNA contour. (D) Intensity profiles along the DNA contour extracted from the original 8-bit image ((A) green line, (B) red line, and (C) blue line). The black line shows the intensity profile obtained from a Cy5-labeled Lambda DNA in the switching buffer containing non-labeled matrix DNA displayed in Figure 4C.

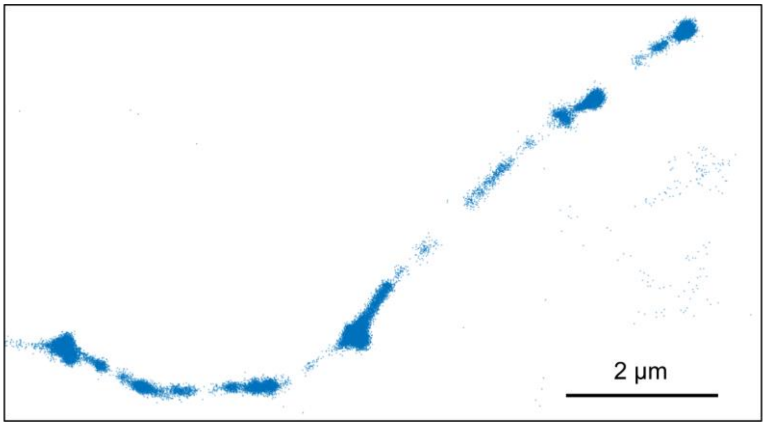

Figure S15. Experimentally obtained SMLM image of a Cy5-labeled Lambda DNA deposited on a glass surface in the switching buffer with the matrix DNA. The image was reconstructed using single-molecule images acquired during 50 seconds and converted to an 8-bit grayscale image.

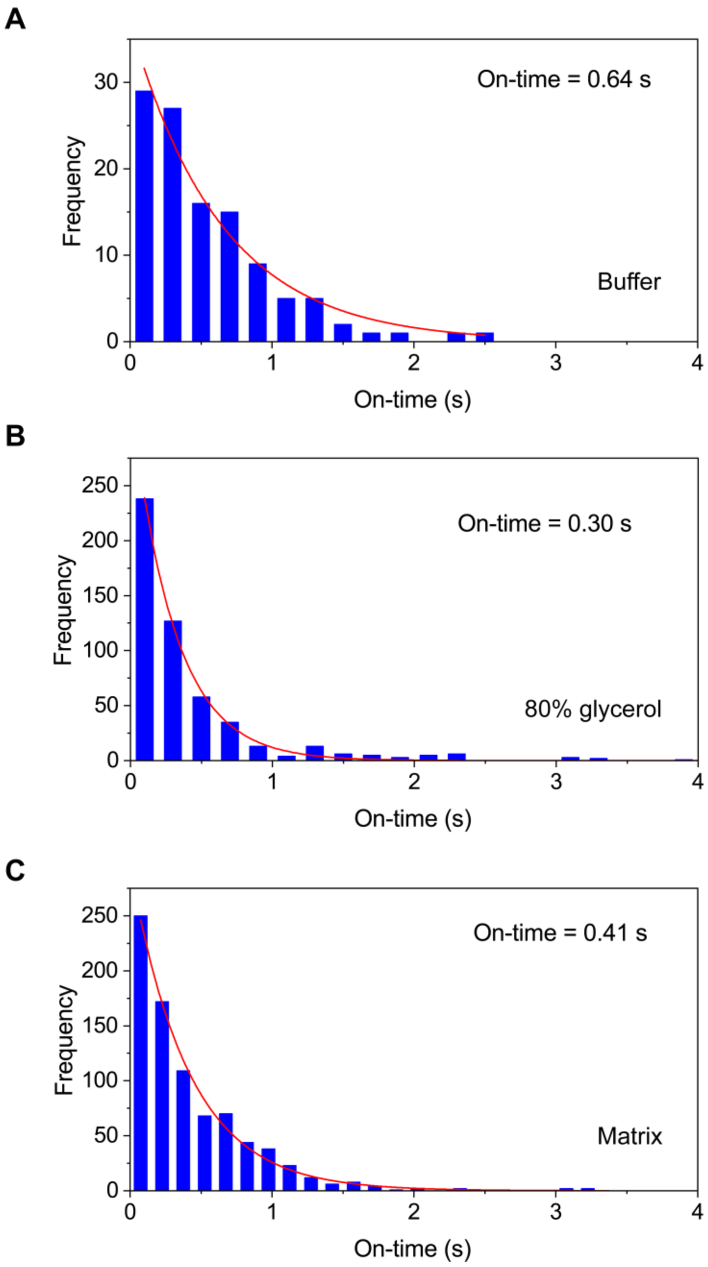

Figure S16. Frequency distributions of the fluorescence on-time of Cy5 conjugated to ssDNA obtained in the switching buffer (A) without glycerol, (B) with 80% glycerol, and (C) with DNA matrix. The red lines show fitting to a single-exponential decaying function.

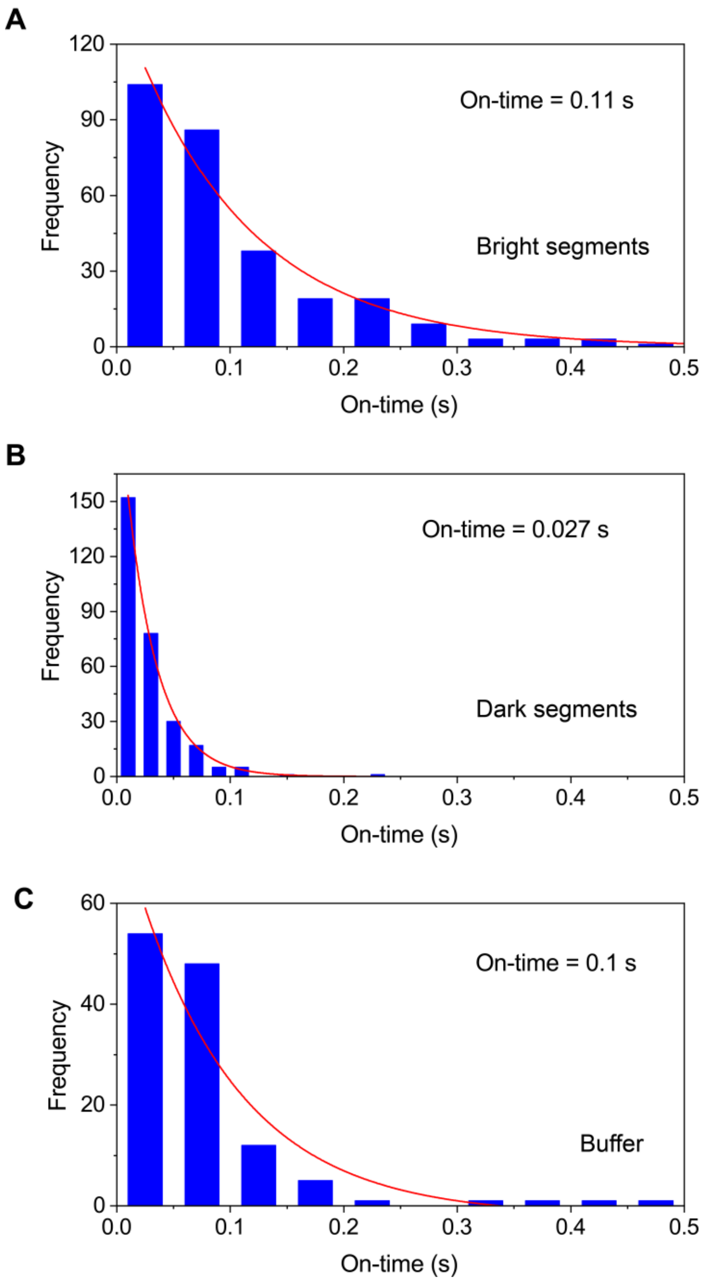

Figure S17. Frequency distributions of the fluorescence on-time of Cy5 conjugated to Lambda DNA in (A) bright and (B) dark segments obtained in the switching buffer with DNA matrix. (C) Frequency distributions of the fluorescence on-time of Cy5 conjugated to Lambda DNA in switching buffer and adsorbed to the glass surface. The red lines show fitting to a single-exponential decaying function.

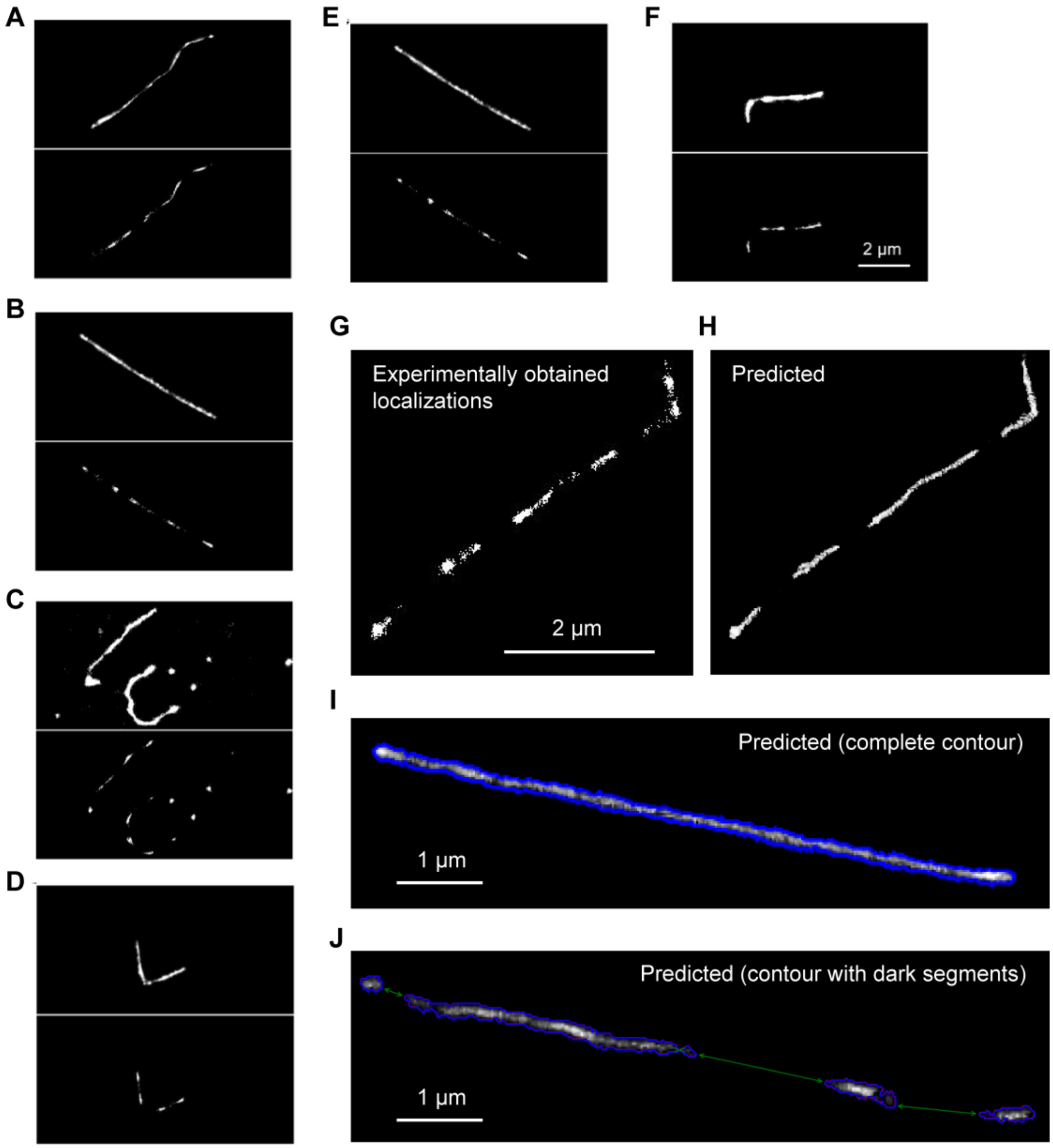

Figure S18. Diverse examples of Lambda DNA contours and localization patterns revealing bright and dark segments. (A-F) Examples of different Lambda DNA molecules imaged using SMLM. For each panel, the upper image shows the 8-bit grayscale representation of all localizations obtained over the entire acquisition time, while the lower image displays a subset of localizations acquired during 1s, highlighting the presence of dark segments. Scale bar = 2 µm. (G) Experimentally obtained SMLM image of Cy5-labeled Lambda DNA, converted to an 8-bit grayscale image. (H) Predicted image for the SMLM image in (G). (I) Predicted image for an SMLM image of Cy5-labeled Lambda DNA, in which the continuous contour highlighted by a blue boundary (referred to as a bright segment) is obtained. (J) Predicted image for an SMLM image of Cy5-labeled Lambda DNA, in which the contour highlighted by a blue boundary is split into multiple segments due to the presence of the regions with a very low localization density (referred to as dark segments) marked with double green arrows.

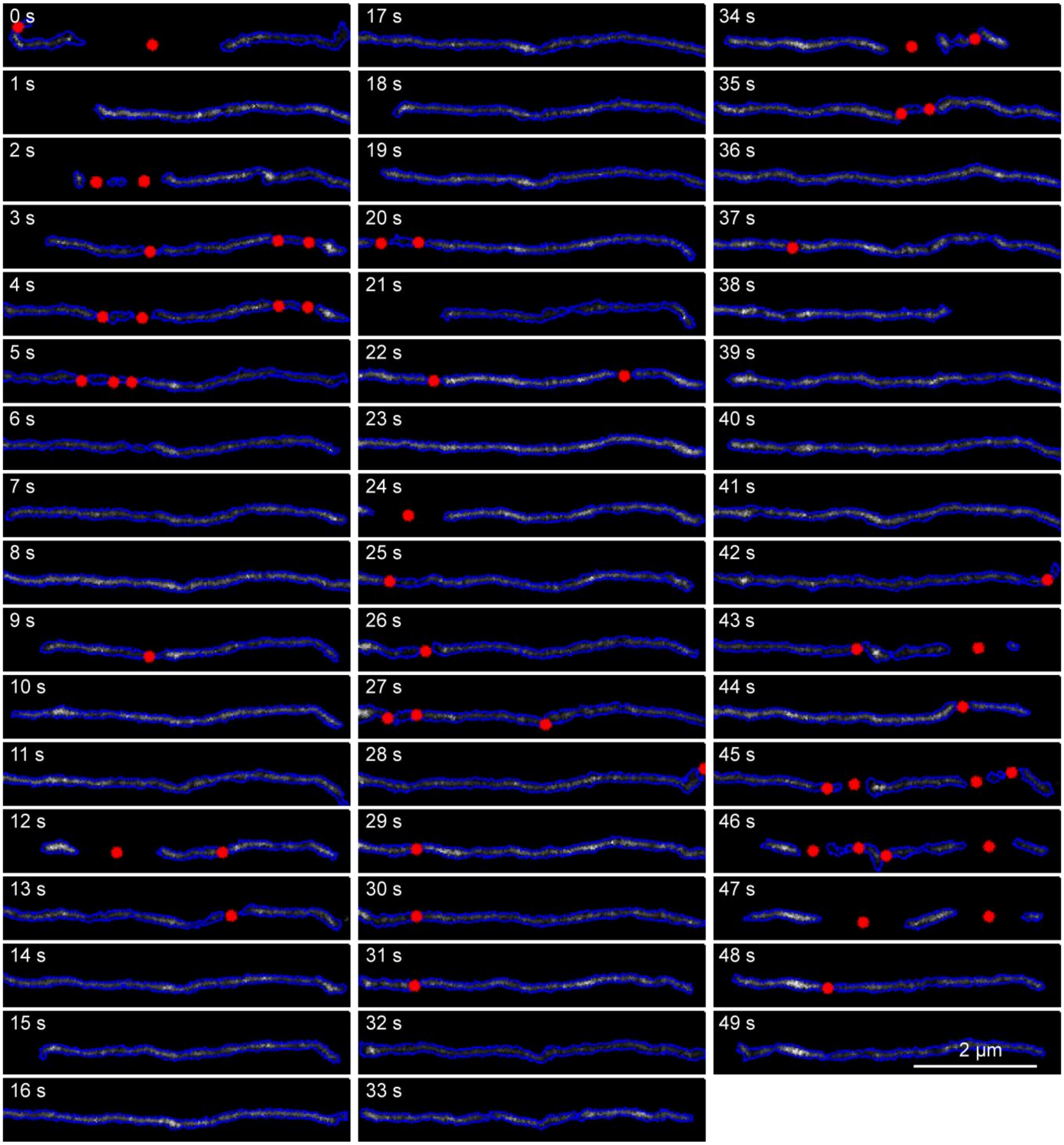

Figure S19. Predicted DNA contours and dark segment centers of Lambda DNA suspended in switching buffer. Scale bar = 2µm.

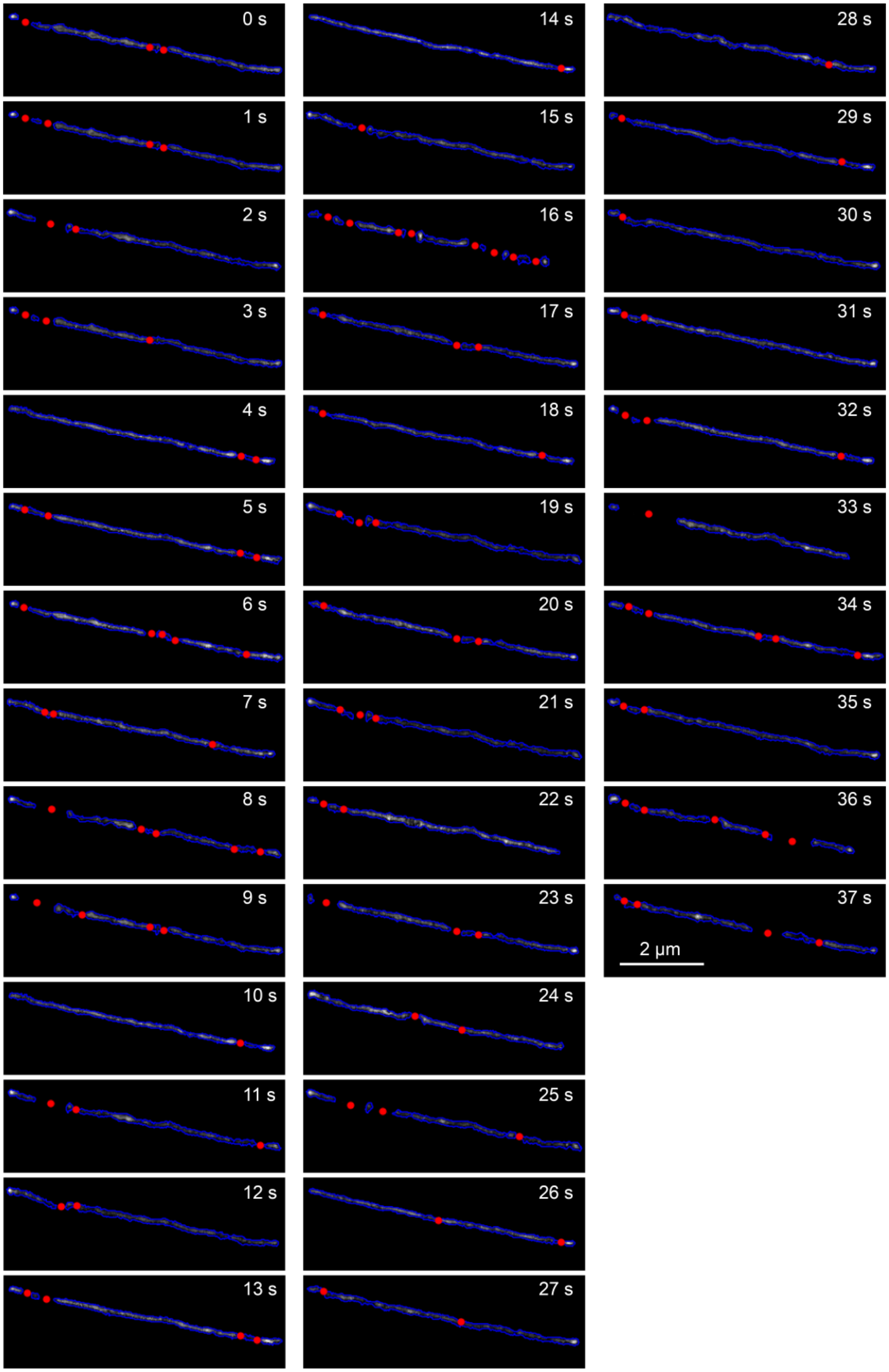

Figure S20. Predicted DNA contours and dark segment centers of Lambda DNA suspended in switching buffer supplemented with 80% glycerol. Scale bar = 2μm.

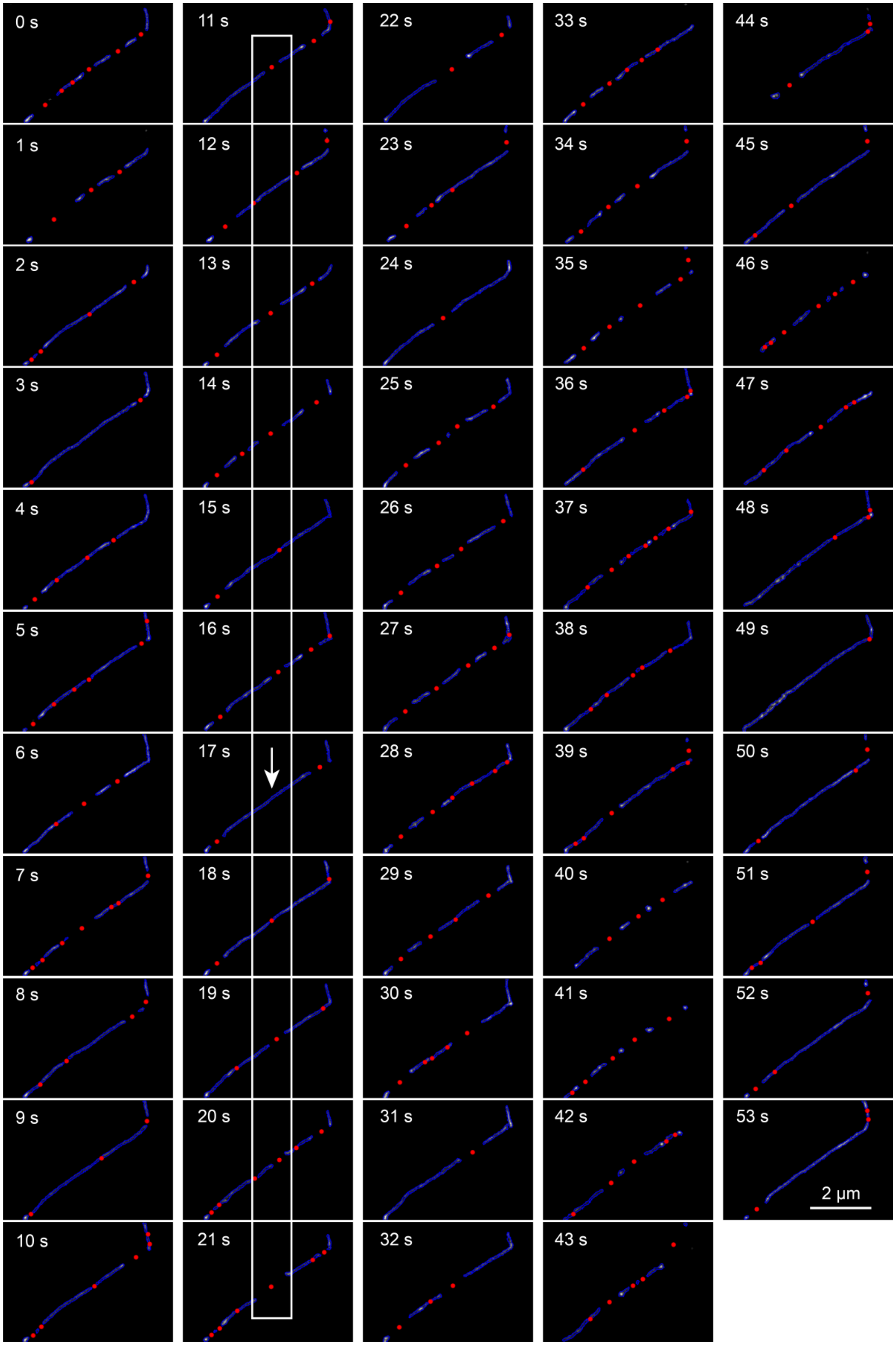
0 s
1 s
2 s
3 s
4 s
5 s
6 s
7 s
8 s
9 s
10 s
11 s
12 s
13 s
14 s
15 s
16 s
17 s
18 s
19 s
20 s
21 s
22 s
23 s
24 s
25 s
26 s
27 s
28 s
29 s
30 s
31 s
32 s
33 s
34 s
35 s
36 s
37 s
38 s
39 s
40 s
41 s
42 s
43 s
44 s
45 s
46 s
47 s
48 s
49 s
50 s
51 s
52 s
53 s
2 μm

Figure S21. Predicted DNA contours and dark segment centers of Lambda DNA suspended in entangled DNA matrix in switching buffer. Scale bar = 2μm. A representative example showing the temporal behavior of dark segments is highlighted by the white rectangle in the second column from 11 – 21 s. While this region exhibits a sustained absence of localizations in most frames, at 17 s (indicated by white arrow), sufficient sparse localizations enable ANNA-PALM to reconstruct the continuous contour. This behavior enabled the calculation of fluorescence on time in these regions (Figure S17B).

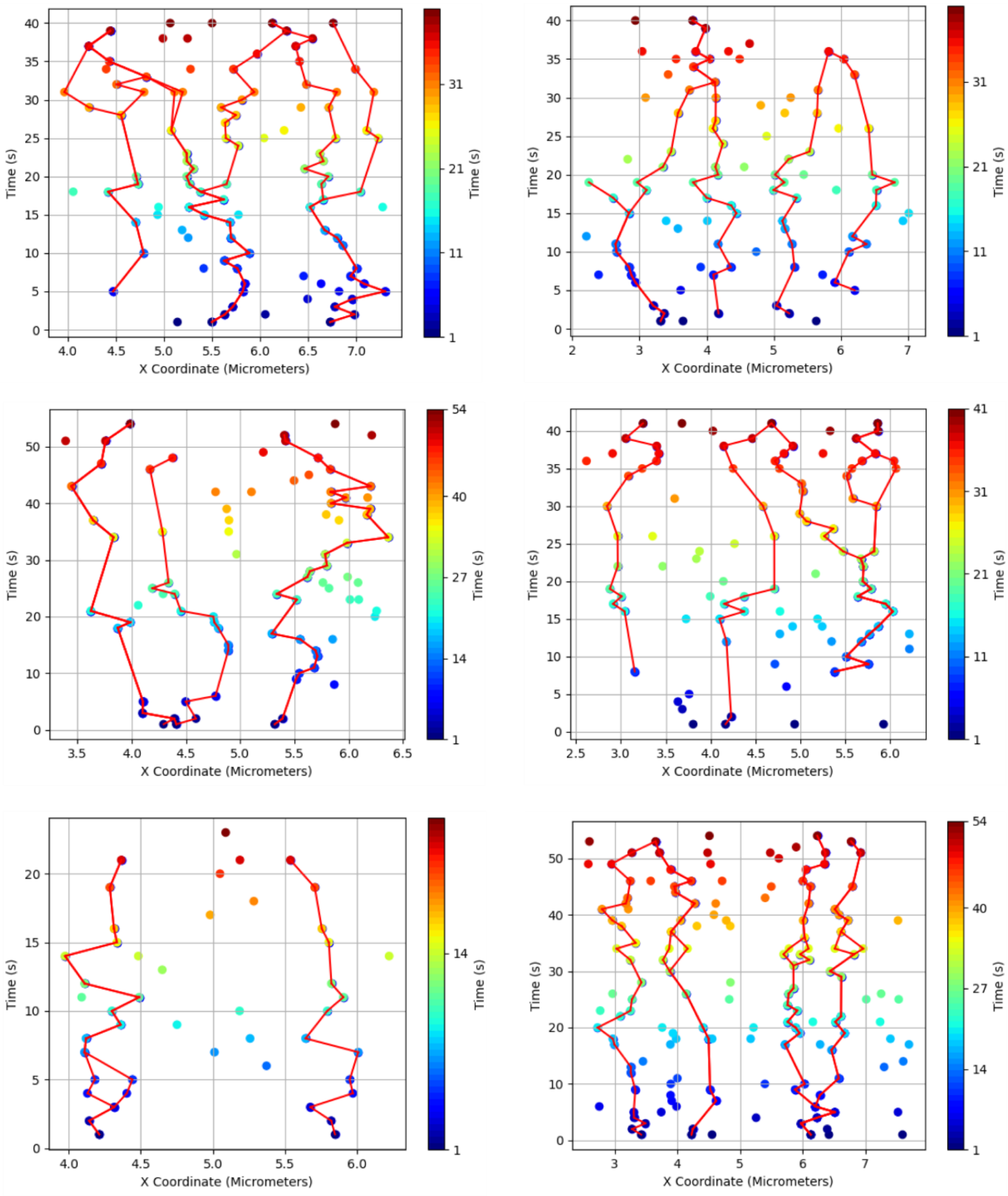

Figure S22. Examples of the temporal dynamics of dark segments' centers of a predicted contour obtained from Lambda DNA in DNA matrix.

tracks.

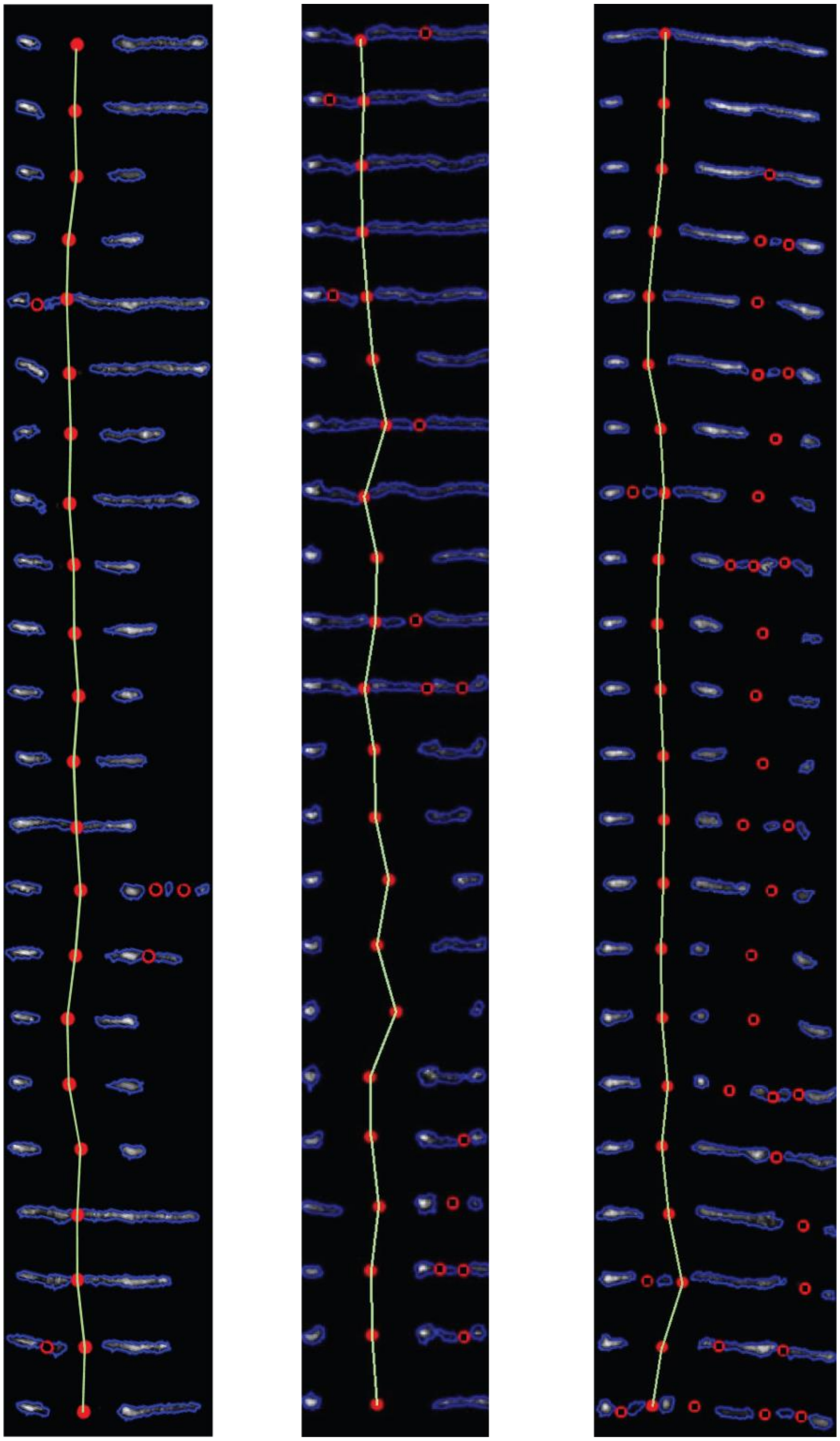

Figure S23. Three examples of the time-lapse images of the predicted contours obtained from Lambda DNA in a DNA matrix with alternating bright and dark segments. The calculated center positions of the dark segments are manually connected with green lines for visualization of the tracks.

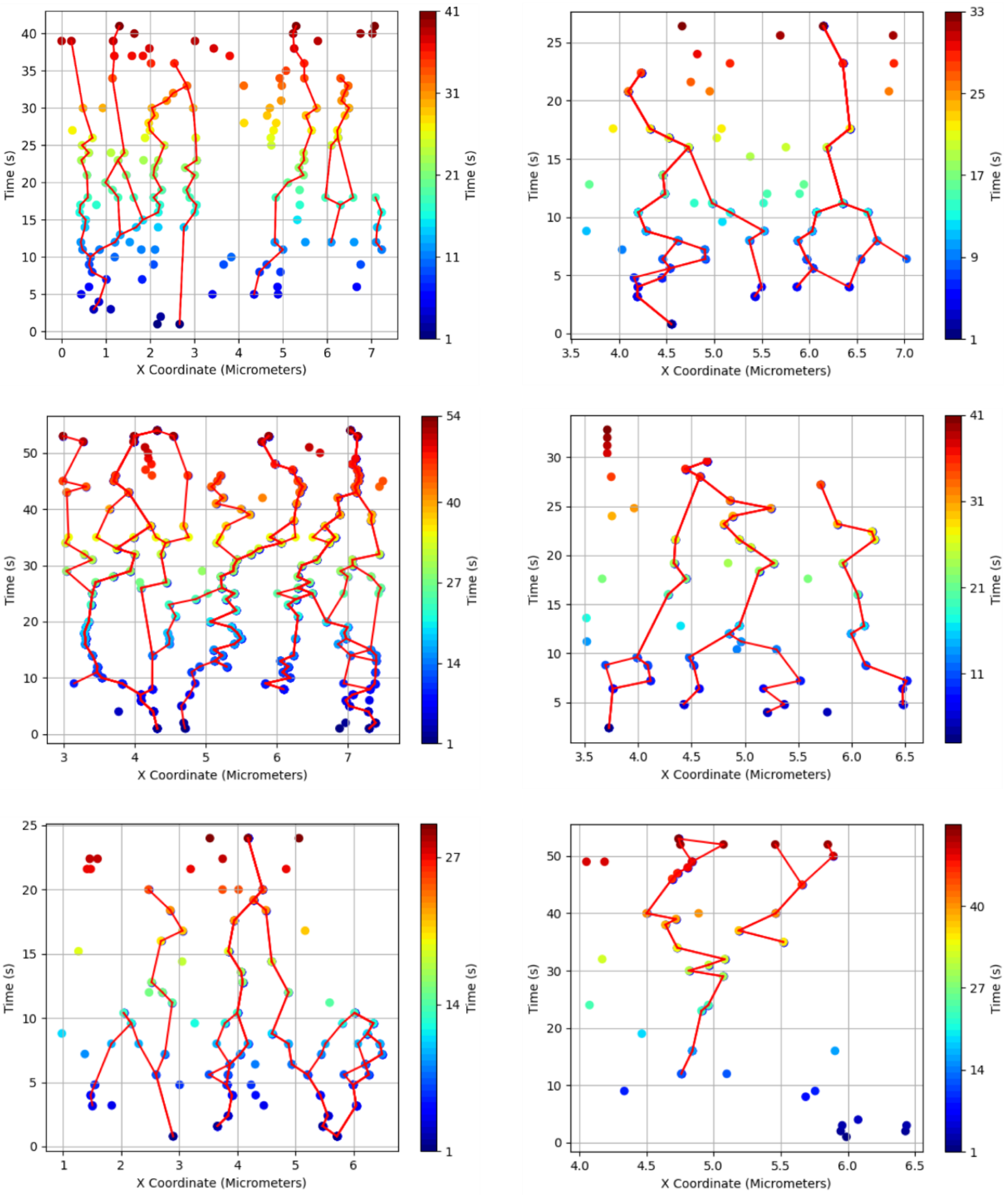

Figure S24. Examples of the temporal dynamics of dark segments' centers of a predicted contour obtained from Lambda DNA suspended in 80% glycerol.

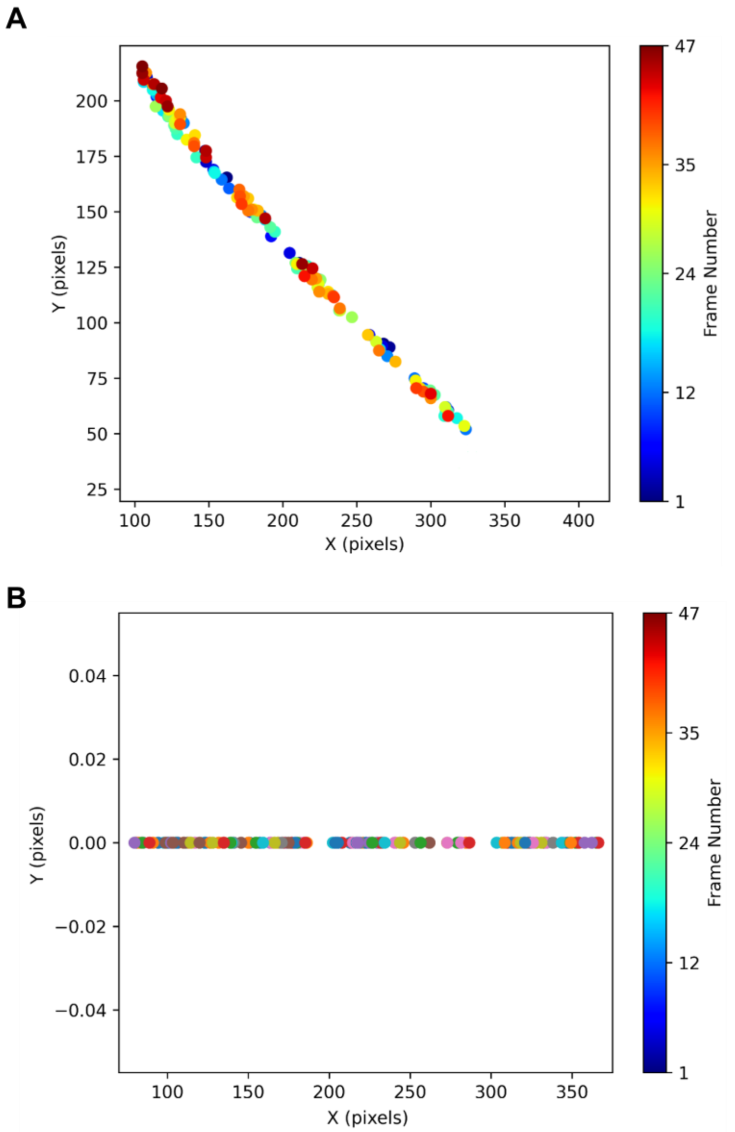

Figure S25. (A) Center positions of the dark segments predicated at different time intervals. Pixel size = 100 nm and integration time = 1 second. (B) The positions of the centers in (A) were rotated relative to the horizontal axis to align horizontally, facilitating the analysis of their dynamics using a one-dimensional random walk model.

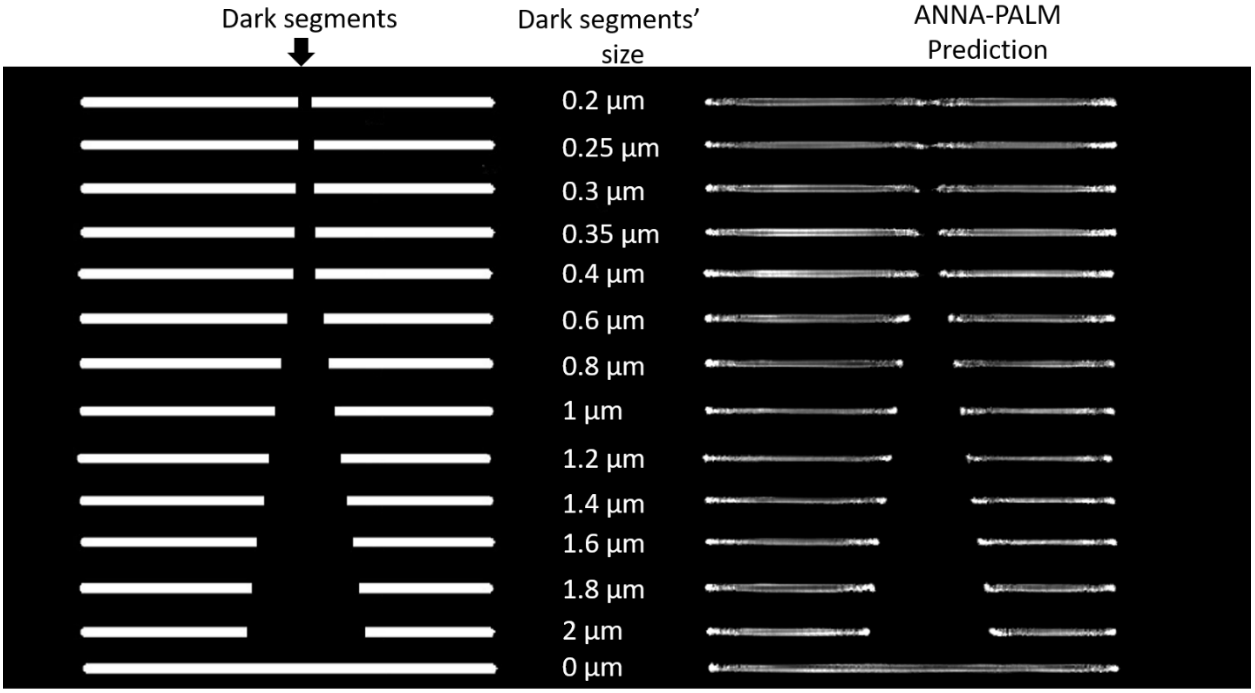

Figure S26. Comparison of dark segment sizes in original and ANNA-PALM predicted images. The dark segments in the original images, manually set with different sizes (left panel), are shown alongside their corresponding ANNA-PALM predicted contours (right panel). The sizes of the dark segments range from 0.2 µm to 2 µm, with 0 µm representing the absence of dark segments. The ANNA-PALM algorithm successfully fills dark segments smaller than 300 nm (0.3 µm), resulting in continuous predicted contours. However, for dark segments larger than 300 nm, the algorithm does not accurately predict the missing contour points, leading to visible gaps in the reconstructed DNA contours.

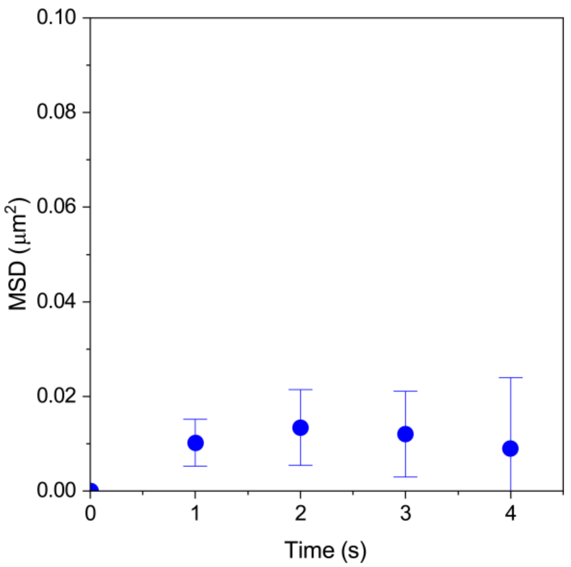

Figure S27. Averaged mean square displacement (MSD) plot versus time lag obtained from the time trajectories of the center positions of the dark segments detected for Lambda DNA suspended in 80% glycerol displayed in Figure S24. The error bars represent the standard deviation of the MSD values obtained in the individual MSD versus time lag plots.

References

(1) J.F. Preston, D.R. Boone, Analytical determination of the buoyant density of DNA in acrylamide gels after preparative CsCl gradient centrifugation, FEBS Letters, 37, 321-324 (1973).

(2) M. Abadi, M.F. Serag, S. Habuchi, Entangled polymer dynamics beyond reptation, Nature Communications, 9, 5098 (2018).

(3) M.E. Sanborn, B.K. Connolly, K. Gurunathan, M. Levitus, Fluorescence Properties and Photophysics of the Sulfoindocyanine Cy3 Linked Covalently to DNA, The Journal of Physical Chemistry B, 111, 1064-11074 (2007).
